# Rho A/ROCK1 signaling-mediated metabolic reprogramming of valvular interstitial cells toward Warburg effect accelerates aortic valve calcification via AMPK/RUNX2 axis

**DOI:** 10.1038/s41419-023-05642-1

**Published:** 2023-02-11

**Authors:** Huiruo Liu, Hang Yin, Zhen Wang, Qiuhuan Yuan, Feng Xu, Yuguo Chen, Chuanbao Li

**Affiliations:** 1 Department of Emergency Medicine and Chest Pain Center, Qilu Hospital of Shandong University, Jinan, Shandong China; 2 Key Laboratory of Emergency and Critical Care Medicine of Shandong Province, Qilu Hospital of Shandong University, Jinan, Shandong China; 3 The Key Laboratory of Cardiovascular Remodeling and Function Research, Chinese Ministry of Education and Chinese Ministry of Public Health, Qilu Hospital of Shandong University, Jinan, Shandong China

**Keywords:** Valvular disease, Calcification

## Abstract

The aberrant differentiation of valvular interstitial cells (VICs) to osteogenic lineages promotes calcified aortic valves disease (CAVD), partly activated by potentially destructive hemodynamic forces. These involve Rho A/ROCK1 signaling, a mechano-sensing pathway. However, how Rho A/ROCK1 signaling transduces mechanical signals into cellular responses and disrupts normal VIC homeostasis remain unclear. We examined Rho A/ROCK1 signaling in human aortic valves, and further detected how Rho A/ROCK1 signaling regulates mineralization in human VICs. Aortic valves (CAVD *n* = 22, normal control (NC) *n* = 12) from patients undergoing valve replacement were investigated. Immunostaining and western blotting analysis indicated that Rho A/ROCK1 signaling, as well as key transporters and enzymes involved in the Warburg effect, were markedly upregulated in human calcified aortic valves compared with those in the controls. In vitro, Rho A/ROCK1-induced calcification was confirmed as AMPK-dependent, via a mechanism involving metabolic reprogramming of human VICs to Warburg effect. Y-27632, a selective ROCK1 inhibitor, suppressed the Warburg effect, rescued AMPK activity and subsequently increased RUNX2 ubiquitin-proteasome degradation, leading to decreased RUNX2 protein accumulation in human VICs under pathological osteogenic stimulus. Rho A/ROCK1 signaling, which is elevated in human calcified aortic valves, plays a positive role in valvular calcification, partially through its ability to drive metabolic switching of VICs to the Warburg effect, leading to altered AMPK activity and RUNX2 protein accumulation. Thus, Rho A/ROCK1 signaling could be an important and unrecognized hub of destructive hemodynamics and cellular aerobic glycolysis that is essential to promote the CAVD process.

## Introduction

Calcific aortic valve disease (CAVD) is an increasingly frequent valvular heart disease in aging populations of the Western world [1], which is characterized by fibro-calcific remodeling of aortic valve leaflets [2]. It is suggested that CAVD is a chronic and progressive disorder that includes aortic valve sclerosis and calcific aortic valve stenosis phases, initiated by risk factors similar to those of atherosclerosis [3]. Currently, there is no effective pharmacology approach to prevent or reduce the progression of CAVD [4], and despite intervention to known risk factors, the therapeutic effects of anti-inflammation or lipid modulation remain unsubstantiated. Current management of CAVD appears to be a wait-and-see schema until severe stenosis presenting with clinically apparent, such as angina and cardiac insufficiency, warranting aortic valve replacement, which comes with complications and no guarantee of long-time survival; however, it is regarded as the only choice for patients with CAVD so far [5].

Valvular calcification was once considered as a passive change of dead and dying cells [6]. Exploration of histological similarities with osteogenesis [7], as well as upregulation of genes encoding bone morphogenetic proteins [8, 9], including runt-related transcription factor 2 (RUNX2), in calcified human aortic valves provided intriguing evidence that vibrantly regulated osteogenic processes participating in valvular calcification. Valvular interstitial cells (VICs) compromise a major component of aortic valves and are active in the production of mineralization of valve leaflets via their abilities for aberrant differentiation to osteogenic lineages, which has been identified as the theoretical basis of the ectopic ossification of aortic valves [10]. Recent studies of pharmacotherapies to treat CAVD have targeted VICs’ osteogenic phenotypic switch in an attempt to halt the progression of CAVD [11, 12]. Exposure of aortic valve leaflets to constantly changeful hemodynamics and high shear stress might trigger mechanosensing-dependent VICs activation and promote pathological differentiation in CAVD.

Ras homolog family member A (Rho A)/Rho associated coiled-coil containing protein kinase 1 (ROCK1) mechanical-sensitive signaling is involved in several physiological processes, such as proliferation, differentiation, migration, and apoptosis, via shrinking or stretching of the cellular cytoskeleton. Interestingly, Rho A/ROCK1 signaling is positively associated with progression and metastasis of cancerous cells [13–19], and Mah et al. reported that ROCK inhibition alleviated breast cancer invasion partially by shifting tumorous cells metabolism from glycolysis to oxidative phosphorylation (OXPHOS) [20]. Furthermore, Rho A/ROCK blocking-up was found abolished mutant p53 (mutp53)-mediated Warburg effect through inhibiting glucose transporter type 1 (GLUT1) translocation to the plasma membrane and consequently compromised mutp53’s tumorigenic function in mouse cells [21]. The Warburg effect (also called aerobic glycolysis), manifested as a preference for glycolysis to generate energy even under adequate oxygen conditions [22, 23], is extensively documented in atherosclerosis and skeletal development, which share similar pathophysiological calcification features to those of CAVD. As the main cells causing valve calcification, Ca/P-induced calcified human VICs also appear an increased glycolytic capacity [24]. Rho A/ROCK1 signaling has been implicated in both osteogenic differentiation of VICs and aortic valve calcification in animal models [25–30]. Nonetheless, the regulatory role of Rho A/ROCK1 signaling in human CAVD models has not been elucidated, additionally the mechanisms underlying any positive effect of Rho A/ROCK1 on CAVD require further study.

AMP-activated protein kinase (AMPK) is a canonical regulator of energy homeostasis, and can sense the metabolic switch in status from OXPHOS to glycolysis. Aberrant levels of AMPK are noted in various pathological settings of calcification, such as osteogenesis and atherosclerosis [31–34], and lower AMPK activity in human calcified aortic valves (CAVs) was detected [35]. AMPK has various biological functions, while its inhibition could adjust RUNX2 protein expression in a ubiquitin-proteasome pathway dependent manner. Therefore, a better understanding of the role of AMPK in VIC osteogenic differentiation might contribute to determining the function of Rho A/ROCK1 in CAVD progression.

In the present study, we explored the possible mechanisms of Rho A/ROCK1 signaling in CAVD, focusing on metabolic reprogramming to the Warburg effect during the osteogenic differentiation of VICs. We found that AMPK received these energy variation signals and then promoted VICs calcification via decreasing ubiquitin-mediated RUNX2 proteasomal degradation. Broadly speaking, this study demonstrated that the Rho A/ROCK1/Warburg effect/AMPK/RUNX2 axis is a critical mechanism to explain the interactions between abnormal hemodynamic forces and VICs calcification, which could become a therapeutic target for CAVD treatment.

## Materials and methods

### Human aortic valves collection

Human aortic valve tissues were obtained in accordance with the Declaration of Helsinki and under informed consent using protocols approved by the ethics committee of Qilu Hospital of Shandong University (*catalog:* KYLL-202111-229-1). In total, 47 patients were screened between April 2022 and July 2022. Five patients were excluded from further analysis because of bicuspid aortic valve morphology, and six were excluded due to a history of rheumatic heart disease. In addition, two patients withdrew after refusing to sign the informed consent form. A total of 34 patients, consisting of 22 patients with CAVD and 12 non-CAVD controls (NC), were finally included in the current analysis. Calcified aortic valve tissues were obtained from 22 patients with CAVD who underwent aortic valve replacement due to severe aortic valve stenosis confirmed by doppler echocardiography and pathology. Non-calcified aortic valve samples were obtained from 12 patients who suffered aortic dissection and received Wheat or Bentall procedures in our institution. All samples were resected aseptically and intraoperatively, without any annular tissues, rinsed with cold sterile phosphate-buffered saline (PBS) (Tianjin TBD Haoyang BioTech Co. Ltd., Tianjin, China) to remove blood components, and preserved in liquid nitrogen or 4% paraformaldehyde (PFA) (Biosharp, Hefei, China) immediately. The baseline demographic characteristics of the CAVD group and the control group are summarized in Table 1.

**Table 1:** Baseline characteristics.

Variables	CAVD (*N* = 22)	NC (*N* = 12)	*P* value
Clinical characteristics			
Age, years	60.1 ± 11.8	61.4 ± 9.7	0.751
Male gender, *n* (%)	14 (63.6)	10 (83.3)	0.432
Medical History			
HP, *n* (%)	5 (22.7)	6 (50.0)	0.138
DM, *n* (%)	2 (9.1)	2 (16.7)	0.602
CAD, *n* (%)	5 (22.7)	5 (41.7)	0.271
Echocardiographic data			
LVEF, %	56.9 ± 13.5	56.8 ± 6.8	0.974
LA diameter, mm	42.5 ± 8.5	49.8 ± 14.7	0.071
LV diameter, mm	52.6 ± 10.6	62.3 ± 11.3	0.018
RV diameter, mm	25.0 ± 3.6	24.4 ± 3.0	0.636
IVST, mm	13.1 ± 2.3	11.5 ± 2.7	0.080
LVPWT, mm	11.4 ± 2.1	10.8 ± 2.1	0.759
AAD, mm	39.1 ± 6.4	40.2 ± 8.5	0.680
MPA diameter, mm	25.6 ± 5.0	27.1 ± 3.0	0.364
maxPG, mmHg	101.0 ± 41.7	25.2 ± 10.6	0.001
meanPG, mmHg	60.5 ± 27.2	12.0 ± 4.6	0.001
Max V, cm/s	492.7 ± 102.0	246.4 ± 47.5	N/A
AOD, mm	22.6 ± 3.2	24.1 ± 4.1	0.304
AVA, cm^2^	0.8 ± 0.3	1.3 ± 0.0	N/A
Laboratory findings			
HDL-C, mmol/L	1.1 ± 0.2	1.2 ± 0.3	0.331
LDL-C, mmol/ L	2.5 ± 0.7	2.2 ± 0.8	0.247
sdLDL-C, mmol/ L	0.6 ± 0.3	0.4 ± 0.2	0.093
Cho, mmol/ L	4.1 ± 0.9	3.9 ± 0.8	0.429
TG, mmol/ L	1.3 ± 0.7	0.9 ± 0.3	0.046
NEFA, umol/dl	46.6 ± 22.2	43.3 ± 18.4	0.665
Cr, umol/L	70.9 ± 12.0	80.2 ± 13.7	0.050
eGFR, ml/min/1.73m^2^	93.1 ± 11.8	86.5 ± 11.3	0.122
GLU, mmol/ L	5.4 ± 2.8	4.6 ± 0.8	0.343
HbA1c, %	5.5 ± 2.9	4.5 ± 0.7	0.226
Calcium, mmol/L	2.3 ± 0.1	2.2 ± 0.1	0.174
Phosphate, mmol/L	1.1 ± 0.2	1.2 ± 0.1	0.610
NT-pro BNP, pg/ml	2165.5 ± 2235.9	1730.4 ± 2431.4	0.708

*CAVD* calcified aortic valve disease, *NC* negative control, *HP* hypertension, *DM* diabetes mellitus, *CAD* coronary artery disease, *LVEF* left ventricular ejection fraction, *LA* diameter left atrium diameter, *LV* diameter left ventricle diameter, *RV* diameter right ventricle diameter, *IVST* interventricular septum thickness, *LVPWT* left ventricular posterior wall thickness, *AAD* ascending aorta diameter, *MPA* diameter main pulmonary artery diameter, *MaxPG* maximum pressure gradient, *MeanPG* mean pressure gradient, *Max V* maximum flow velocity of aortic valve, *AOD* aortic diameter, *AVA* aortic valve orifice area, *HDL-C* high-density lipoproteincholesterol, *LDL-C* low-density lipoproteincholesterol, *sdLDL-C* small dense low-density lipoproteincholesterol, *Cho* cholesterol, *TG* triacylglycerol, *NEFA* non-esterified fatty acid, *Cr* creatinine, *eGFR* estimated glomerular filtration rate, *GLU* blood glucose, *HbA1c* glycosylated hemoglobin Alc, *NT-proBNP* N-terminal pro brain natriuretic peptide.

Data are presented as mean ± SD or absolute numbers (proportions).

### Cell isolation and culture

Primary human VICs were isolated using a previously described method with modifications [36]. Briefly, surgically obtained human aortic valve leaflets were washed with cold PBS (containing 1% penicillin/streptomycin (P/S) (Gibco, Grand Island, NY, USA)), and then cut into approximately 3 x 3 mm pieces. Next, valve leaflets were subjected to digestion with 2 mg/ml collagenase II (Worthington Biochemical Corporation, Lakewood, NJ, USA) and incubated at 37 °C for 30 min, and then shaken vigorously to remove any remaining valvular endothelial cells (VECs). Valve samples were then washed in sterile PBS again and incubated in 2 mg/ml collagenase II for 2 h at 37 °C. The resulting cells were collected by centrifugation at 12000 rpm for 5 min. Human primary VICs were seeded in T25 flasks or plates and cultured in high glucose Dulbecco’s modified Eagle’s medium (DMEM) (Gibco, containing 2 mM L-glutamine, 4.5 g/L D-glucose and 110 mg/L sodium pyruvate) supplemented with 10% fetal bovine serum (FBS) (Gibco, 10100139 C) and 1% P/S, and stimulated for the indicated times. Human VICs were maintained in an incubator containing 5% CO2 at 37 °C. VICs in all in vitro experiments were utilized from passages 2 to 7.

### Osteogenic differentiation of human VICs

To stimulate calcification, human VICs were cultured with inorganic phosphate-osteogenic medium (IP-OM) (complete media supplemented with 2 mM sodium dihydrogen phosphate (Beyotime Biotech, Jiangsu, China), 75 mM ascorbic acid (Beyotime Biotech), 100 nM dexamethasone (Beyotime Biotech) and 10^−7^ mM insulin (Beyotime Biotech)) for 7–9 days, modified from previously described approaches [4, 37, 38]. Calcific nodules could be observed by day 3. The osteogenic differentiation medium was changed every 2 days.

### Alizarin red staining

For human VICs, following treatments, cells were washed three times with 1x PBS to remove medium, then fixed in 4% PFA (Biosharp) for 30 min at room temperature, and rinsed with double distilled water. Fixed VICs were then stained with 2% alizarin red stain (pH-4.2) (Beyotime Biotech) in the dark, overnight at room temperature. VICs were next washed three times with 1x PBS or double distilled water to remove non-specific staining. Mineralized nodules stained with Alizarin Red manifested as red depositions. For human aortic valve tissues, paraffin-embedded sections (5 μm) were prepared, then twice deparaffinized using dewaxed solution, and hydrated with ethyl alcohol (SCR) using a concentration gradient (100%, 90%, 80%, 70%, 60%). The tissue sections were then incubated with Alizarin Red solution for 2 h and washed in water to remove excessive dye. For quantification, images were photographed using a microscope (CKX53, Olympus, Tokyo, Japan) and captured using CellSens software (Olympus). Then Alizarin Red positive areas were assessed using Image J analysis (NIH, Bethesda, MD, USA) and the mean was determined from three independent biological replicates.

### Alkaline phosphatase staining

Alkaline phosphatase (ALP) staining was processed per the manufacturer’s instructions of the 5-bromo-4-chloro-3-indolyl-phosphate (BCIP)/nitro blue tetrazolium (NBT) Alkaline Phosphatase Color Development Kit (Beyotime Biotech). Human VICs were fixed in 4% PFA for 30 min at room temperature after washing in 1x PBS supplemented with Tween20 (Biosharp) (PBST). This was followed by three washes with PBST and then the cells were incubated with moderate BCIP/NBT dyeing solution at room temperature for 1 to 2 h protected from light until the desired coloration displayed. VICs were next rinsed with 1x PBS three times. Images were acquired under a microscope (CKX53, Olympus). ALP-positive areas were calculated using Image J software, and the mean was determined from three independent biological replicates.

### RNA isolation and quantitative real-time reverse transcription PCR

Total RNA was isolated from human VICs using the Trizol Reagent (Thermo Fisher Scientific, Waltham, MA, USA) following the manufacturer’s protocol. Two micrograms of total RNA were reversely transcribed into cDNA using M-MLV Reverse Transcriptase (Invitrogen, Waltham, MA, USA). Then, quantitative real-time PCR was performed using SYBR Green chemistry to examine the mRNA expression level of *RUNX2*. The housekeeping gene, *ACTB* (encoding β-actin) was used to normalize the differences in the efficiencies of reverse transcription. The primer sequences are listed in Supplemental Table S2.

### Western blotting

Western blotting analysis was applied to examine protein levels using antibodies recognizing Rho A (Abcam, Cambridge, MA, USA), ROCK1 (Abcam), ROCK2 (Abcam), myosin phosphatase-targeting subunit 1 (MYPT1) (Cell Signaling Technology (CST), Danvers, MA, USA), phosphorylated (p)-MYPT1 (CST), glucose transporter type 1, erythrocyte/brain (GLUT1) (Abcam), hexokinase 2 (HK2) (Abcam), pyruvate dehydrogenase kinase 1(PDK1) (Abcam), phosphofructokinase 1 (PFK1) (CST), lactate dehydrogenase A (LDHA) (Abcam), osteopontin (OPN) (Abcam), RUNX2 (Abcam), AMPK (Abcam), p-AMPK (Abcam), ubiquitination (Proteintech, Rosemont, IL, USA), Sodium Potassium ATPase (Abcam), and β-actin (Proteintech). Human aortic valve tissues and VICs were rinsed with cold 1× PBS, and then lysed on ice in radioimmunoprecipitation assay (RIPA) lysis buffer (Beyotime Biotech; 50 mM Tris, pH 7.4, 150 mM NaCl, 1% Triton X-100, 1% sodium deoxycholate, 0.1% sodium dodecyl sulfate (SDS), 1 mM sodium orthovanadate, 1 mM sodium fluoride, 1 mM Ethylenediaminetetraacetic acid (EDTA), and leupeptin) containing 1 mM phenylmethylsulfonyl fluoride (PMSF) for 15 min, followed by homogenization with 20 kHz ultrasonic lapping and centrifugation at 14,000 rpm at 4 °C in a refrigerated microcentrifuge for 15 min to extract total protein. Then, supernatants were stored and a bicinchoninic acid (BCA) Protein Assay Kit (Beyotime Biotech) was used to detect the total protein concentration to normalize the samples. Equal amounts of samples (30–45 μg/lane) were run on sodium dodecyl sulfate-polyacrylamide gel electrophoresis (SDS-PAGE) gels and then transferred to polyvinylidene difluoride (PVDF) membranes using a wet-transfer system. The membranes were blocked using QuickBlock Blocking Buffer (Beyotime Biotech) at room temperature for 20 min, and then incubated with primary antibodies at 4 °C overnight. After three washes with Tris-buffered saline-Tween-20 (TBST) (Servicebio, Wuhan, China), we incubated membranes with the corresponding secondary antibodies coupled with horseradish peroxidase (HRP) for 90 min on shakers. After three washes with TBST, enhanced chemiluminescence (ECL) signals (Amersham Biosciences, Piscataway, NJ, USA) were detected using an ECL kit (Millipore, Billerica, MA, USA) with Imaging system (Thermo Fisher Scientific). Densitometric quantification was performed using Image J software.

### Ubiquitination assay

Human VICs were treated with the proteasome inhibitor MG132 at 100 nM for 15 h before harvest. Cell lysates were immunoprecipitated using anti-RUNX2 antibodies, and then analyzed by immunoblotting using anti-Ubiquitin antibodies.

### Measurements of adenosine levels

Human VICs were homogenized with 20 kHz ultrasonic lapping and then incubated in boiled Tris-Acetate buffer for 90 s to extract adenosine. Levels of ATP, ATP + ADP, and ATP + AMP were detected using an AMP-Glo™ Assay (Promega, Madison, WI, USA) according to the manufacturer’s instructions. Cellular contents of adenosine were normalized to the total protein content.

### Immunohistochemistry

Immunohistochemistry detection of Rho A, ROCK1, ROCK2, GLUT1, HK2, PDK1, PFK1, LDHA, OPN and RUNX2 in human aortic valve leaflets was performed. 4% PFA-fixed paraffin sections (5μm thick) were deparaffinized using dewaxed solution and hydrated using decreasing concentration of ethyl alcohol (100%, 90%, 80%, 70%, 60%), and then incubated at 65 °C for 20 min in antigen unmasking solution using PT Module-Lab Vision (Thermo Fisher Scientific) for antigenic retrieval. Following several washes (3 x 5 min) in PBS, paraffin sections were blocked for 30 min using 5% bovine serum albumin (BSA). After blocking, primary antibodies against Rho A (abcam, ab54835), ROCK1 (abcam, ab97592), ROCK2 (abcam, ab125025), GLUT1 (abcam, ab115730), HK2(abcam, ab209847), PDK1 (abcam, ab202468), PFK1 (CST, #8164), LDHA (abcam, ab52488), OPN (abcam, ab63856) and RUNX2 (abcam, ab192256) were diluted at optimized concentrations in 1% BSA and incubated with the sections in a wet box overnight at 4 °C. Subsequently, appropriate secondary antibodies were applied for 30 min at room temperature. Following secondary antibody incubation, the positive staining was detected using an UltraSensitive SP IHC Kit and DAB Kit, and hematoxylin staining was used for nuclear counterstaining. Images were visualized using a microscope (CKX53, Olympus), and Image J software was applied for data quantification.

### Alkaline phosphatase activity assay

The alkaline phosphatase activity assay was performed according to the manufacturer’s instructions (Beyotime Biotech). Human VICs were harvested and lysed with RIPA lysis buffer without phosphatase inhibitor. Following homogenization and centrifugation, the supernatants were collected. Then supernatants were added with dilution buffer (Beyotime Biotech), p-nitrophenol (Beyotime Biotech) and the chromogenic substrate (Beyotime Biotech), next mixed using a horizontal shaker, and incubated at 37°C for 20 min. The absorbance at 405 nm was detected using an EPOCH2 microplate reader (Thermo Fisher Scientific) to measure alkaline phosphatase activity.

### L-lactate assay

The L-Lactate assay was performed according to the manufacturer’s instructions (Abcam). Briefly, human VICs were harvested, washed with cold PBS, and then resuspended in 4x volumes of Lactate Assay Buffer. After centrifugation for 2 min at 4 °C at top speed in a microcentrifuge to remove insoluble material, the supernatants were collected. LDH was removed from the samples by performing Perchloric acid (PCA)/KOH deproteinization. Then, we detected absorbance at 450 nm using the EPOCH2 microplate reader and constructed a standard curve. The lactate concentration was estimated by plotting on the standard curve.

### Glucose uptake colorimetric assay

The glucose uptake colorimetric assay was performed according to the manufacturer’s instructions (Sigma, St. Louis, MO, USA). Briefly, human VICs were seeded at 1500 cells per well in 96-well plates after appropriate stimulation and incubated in an incubator containing 5% CO2 at 37 °C overnight. VICs were then rinsed twice with sterile PBS and starved in serum-free medium overnight. After another several washes, VICs were glucose-starved in Krebs-Ringer phosphate HEPES buffer containing 2% BSA for 40 min, followed by stimulation with or without insulin for 20 min. VICs were then cultured with 1 mM of the fluorescent glucose analog 2-Deoxy-d-glucose (2-DG) for 20 min. Following incubation, the cells were washed three times with PBS and then lysed using 80 μl of extraction buffer per well. After heating at 85 °C for 40 min, the cell lysates were incubated with Reaction Mix A and Reaction Mix B on a horizontal shaker. Fluorescence was calculated using absorbance at 485 nm in the EPOCH2 microplate reader (Thermo, EPOCH2).

### Glycolysis measurements

The real-time oxygen consumption rate (OCR) and extracellular acidification rate (ECAR) of human VICs were measured using a Seahorse XFe96 flux analyzer (Agilent Technologies, Santa Clara, CA, USA). Briefly, human VICs were seeded into XF96 V3 PS cell culture microplates (Agilent Technologies) at 5000 cells/well and treated with appropriate medium at 37 °C in a CO_2_ incubator, meanwhile hydrated a sensor cartridge at 37 °C in a non-CO_2_ incubator overnight. On the day of real-time ATP production analysis, VICs were rinsed once with sterile Seahorse XF DMEM Medium (pH 7.4) which had been pre-heated to 37 °C (supplemented with 10 mM XF glucose, 1 mM XF pyruvate, and 2 mM XF glutamine). Then, cells were incubated in the above-prepared assay media in a CO_2_-free incubator at 37 °C for 45 min. After this incubation, the assay medium was removed and replaced with fresh warm assay medium in each well. Microplates were placed in the Seahorse XFe96 flux analyzer. ECAR of human VICs was measured in response to 0.5 μM Rotenone/Antimycin A and 50 mM 2-DG using Agilent Seahorse XF Glycolytic Rate Assays, and OCR was measured in response to 1.5 μM Oligomycin, 2 μM FCCP and 0.5 μM Rotenone/Antimycin A using Agilent Seahorse XF Cell Mito Stress Test Kit, according to manufacturer’s instructions. Three/Four calculations were performed under basal conditions and after each drug injection, and relevant metabolic parameters could be calculated using manufacturer-supplied equations.

### Calcium colorimetric assay

The calcium colorimetric assay was performed following the manufacturer’s instructions (Beyotime Biotech). Briefly, human VICs collected after corresponding stimulations were lysed using lysate buffer. The chromogenic reagent and calcium assay buffer were mixed with cell lysates, followed by 5–10 min incubation at room temperature protected from light. The absorbance at 575 nm was then determined using the EPOCH2 microplate reader and a standard curve was established. Finally, the calcium content was calculated according to the standard curve.

### Statistical analysis

All statistical analyses and generation of graphs were performed using GraphPad Prism version 9.0 (GraphPad Inc., La Jolla, CA, USA). Continuous data were expressed as the mean ± the standard deviation (SD), and categorical variables were expressed as absolute numbers and proportions. All experiments were biologically replicated at least three times. In vitro, experiments were performed independently in triplicate. In vivo, western blotting was performed once in statistically relevant group sizes of 22 human aortic valve tissues in CAVD group and 12 in control group. The normality of the distribution of continuous variables was confirmed by the Shapiro–Wilk normality test and was visualized by a Q-Q plot. The homoscedasticity was confirmed by *F*-test. For continuous variables, on the basis of the normal distribution and similar variances, unpaired two-tailed Student’s *t*-test (two groups) and one-way ANOVA followed by Bonferroni post hoc test (≥3 groups) were used; if variances differed between the groups, Welch ´s correction was used; if data showed significantly abnormal distribution, Mann–Whitney *U* test (two groups) and Kruskal–Wallis test followed by Dunn’s post hoc test (≥3 groups) were used. For categorical variables, Pearson’s χ2 or Fisher’s exact test was used. Any p values that were less than 0.05 were considered statistically significant (***p < 0.001, **p < 0.01, or *p < 0.05).

## Results

### Baseline characteristics of study population

The baseline clinical characteristics of the study population are listed in Table 1. A total of 34 patients, consisting of 22 patients with CAVD and 12 non-CAVD controls, were included in the current analysis. Patients were between 27 and 75 years old. Among, the average age was 60.1 ± 11.8 for patients with CAVD compared with 61.4 ± 9.7 for non-CAVD patients (*P* = 0.751). Patients with and without CAVD were predominantly male (63.6% and 83.3%, respectively; *P* = 0.432). Patients with CAVD had similar rates of hypertension (*P* = 0.138), diabetes mellitus (*P* = 0.602), and coronary heart disease (*P* = 0.271) compared with those in the control group. Notably, the CAVD group showed a significantly higher baseline pulmonary valve mean/max pressure gradient (60.5/101.0 *vs*. 12.0/25.2 mmHg, *P* < 0.01 for all), while the control group had an apparent expansion of the left ventricle (52.6 ± 10.6 *vs*. 62.3 ± 11.3 mm, *P* = 0.018), which was related to the pathophysiological diagnosis of severe aortic regurgitation in the majority of patients in the NC group. No statistical significance was found for the left atrium diameter (*P* = 0.071), right ventricle diameter (*P* = 0.636), interventricular septum thickness (*P* = 0.080), aortic diameter (*P* = 0.304), main pulmonary artery diameter (*P* = 0.364), or ascending aorta diameter (*P* = 0.680). Baseline left ventricular ejection fraction (56.9 ± 13.5% *vs*. 56.7 ± 6.8%, *P* = 0.974) and N-terminal pro B-type natriuretic peptide (NT-proBNP) (2165.6 l ± 2235.9 pg/ml *vs*. 1730.4 ± 2431.4 pg/ml, *P* = 0.708), two indexes that are always used to assess cardiac function in clinical practice, were similar between the groups. Aortic valve maximum velocity was obviously higher and aortic valve orifice area was much smaller for patients with CAVD; however, these data obtained were inadequate to perform meaningful analysis. Serological analysis demonstrated significantly higher levels of serum triglyceride for patients with CAVD (1.3 ± 0.7 *vs*. 0.9 ± 0.3 mmol/L, *P* = 0.046), whereas no statistically significant difference was found for high density lipoprotein cholesterol, low density lipoprotein cholesterol, total cholesterol, non-esterified fatty acid, creatinine, estimated glomerular filtration rate, glucose, HbA1c, calcium, or phosphate between the CAVD and control groups (*P* > 0.05 for all).

### Rho A/ROCK1, but not ROCK2, are elevated in human calcified aortic valves and human calcified VICs

**Fig. 1:**
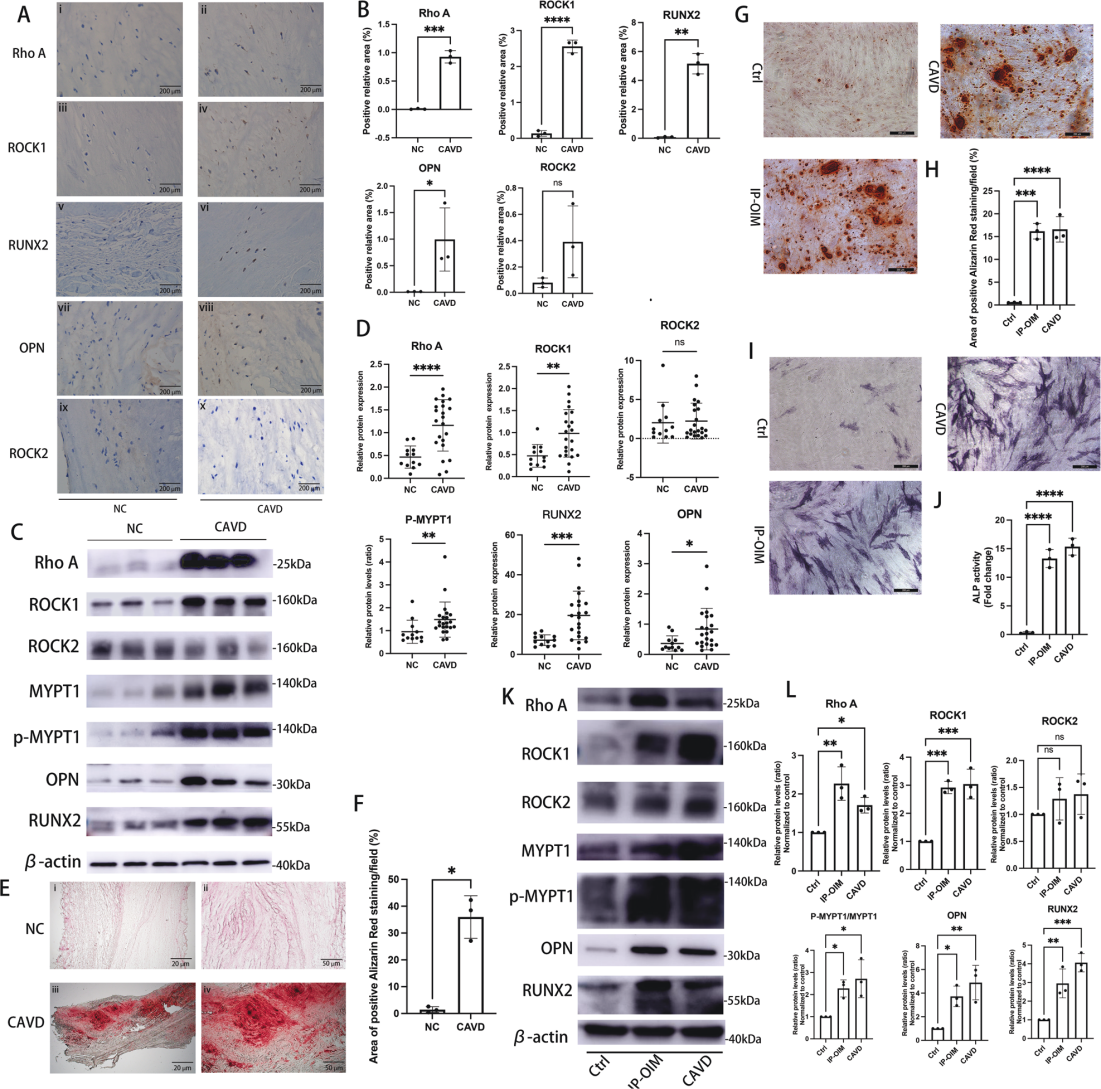
Rho A/ROCK1 signaling are elevated in human calcified aortic valves and human calcified VICs. **A** Immunohistochemistry and (**B**) quantifications for Rho A (brown), ROCK1 (brown), ROCK2 (brown), OPN (brown) and RUNX2 (brown) of human aortic valves from CAVD and NC groups. (Unpaired two-tailed Student’s *t*-test with Welch’s correction for OPN, RUNX2 and ROCK2; unpaired two-tailed Student’s *t*-test for ROCK1 and Rho A). Scale bar = 200 µm. **C** Western blotting analysis for Rho A, ROCK1, ROCK2, MYPT1, p-MYPT1, OPN and RUNX2 of human aortic valves, either calcified (*n* = 22) or non-calcified (*n* = 12) with (**D**) quantifications (Mann–Whitney test for ROCK2, pMYPT1/MYPT1 and OPN; unpaired two-tailed Student’s *t*-test with Welch’s correction for Rho A and RUNX2; unpaired two-tailed Student’s *t*-test for ROCK1). β-actin was used as loading control. **E** Low and high magnification of alizarin red staining of aortic valves from CAVD and NC groups and (**F**) quantifications of positive alizarin red areas/fields (unpaired two-tailed Student’s *t*-test with Welch’s correction). Scale bar = 50 µm (high-power views) and 20 µm (low-power views). **G** Representative alizarin red staining (red) and (**H**) quantifications of human VICs isolated from non-calcified and calcified aortic valves, and human VICs from IP-OIM (one-way ANOVA followed by Bonferroni post hoc test). Scale bar = 200 µm. **I** Representative ALP staining (purple) and (**J**) quantifications of human VICs isolated from non-calcified and calcified aortic valves, and human VICs from IP-OIM (Welch’s ANOVA test). Scale bar = 200 µm. **K** Western blotting and (**L**) quantifications for Rho A, ROCK1, ROCK2, MYPT1, p-MYPT1, OPN and RUNX2 of human VICs isolated from non-calcified and calcified aortic valves, and human VICs from IP-OIM (one-way ANOVA followed by Bonferroni post hoc test). β-actin was used as loading control. Data are shown as mean ± SD. *P < 0.05, **P < 0.01, ***P < 0.001, ****P < 0.0001.

To study Rho A/ROCK signaling in human model of CAVD, we first examined protein expressions of Rho A, ROCK1 and ROCK2 in histologically calcified and non-calcified human aortic valve leaflets. Higher expressions of Rho A and ROCK1 were detected within calcific lesions compared to adjacent non-calcified regions in aortic valves from patients with CAVD, while extensively lower expressions were verified in the controls (Fig. 1A i, ii, iii and iv, and 1B). By contrast, ROCK2 protein expressions were similar in calcified and non-calcified human aortic valves and were low in both groups (Fig. 1A ix and x, and 1B). Consistently, western blotting of aortic valves obtained from 34 patients showed increased protein expressions of Rho A and ROCK1, and phosphorylation levels of MYPT1 in the CAVD group, but not ROCK2 (Figs. 1C and 1D). Calcium-rich regions on aortic valve surface are ectopic osteogenic structures, as demonstrated by alizarin red staining (Fig. 1E and F), and upregulated expressions of osteogenic markers, RUNX2 and OPN, were likewise observed in aortic valves with prominent calcified lesion formation (Fig. 1A to D). We, therefore, isolated VICs from human calcified and non-calcified valves, and examined Rho A, ROCK1 and ROCK2 protein expressions, as well as ROCK activity in normal VICs, normal VICs treated with inorganic phosphorus-osteogenic medium (IP-OM), and diseased VICs extracted from CAVs. Human VICs from calcifying valves, as well as VICs following 7-day OM-treatment exhibited an osteogenic phenotype, characterized by increased alizarin red (Figs. 1G and 1H) and alkaline phosphatase reactivity (Figs. 1I and 1J), and upregulation of calcification markers, OPN and RUNX2 (Figs. 1K and 1L). Rho A, ROCK1 protein expressions and phosphorylation levels of MYPT1 were markedly increased in both VICs isolated from CAVs and VICs undergoing OM-induced-calcification (Figs. 1K and 1L). However, ROCK2 protein levels were similar to those in non-calcified human VICs (Figs. 1K and 1L).

### IP-induced human VICs osteogenic induction model in vitro

**Fig. 2:**
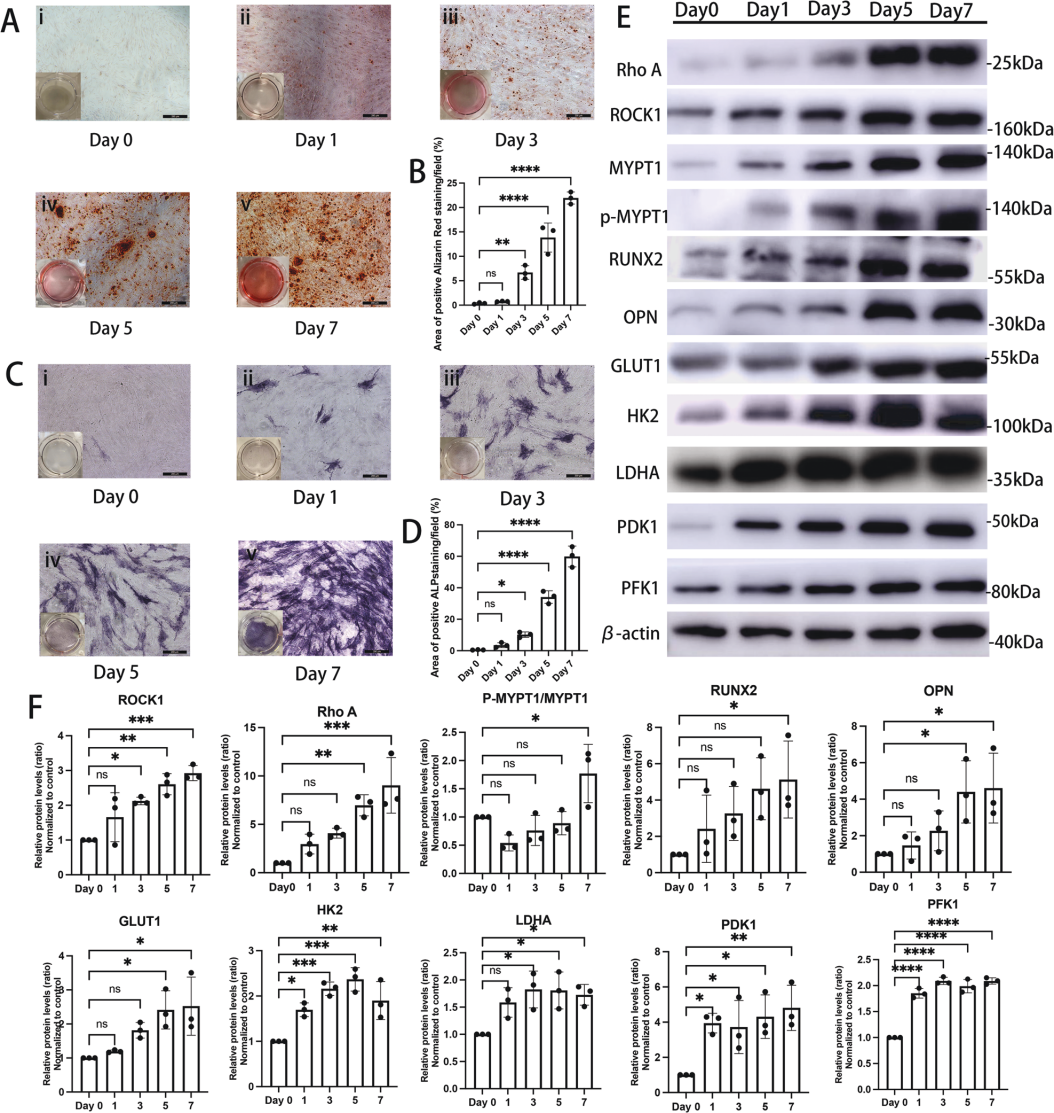
IP-induced osteogenic differentiation of human VICs in vitro. **A** Alizarin red staining of human VICs under osteogenic stimulus for 0 (i), 1 (ii), 3 (iii), 5 (iv) and 7 (v) days. Scale bar = 200 µm. **B** Quantifications of data presented in A (one-way ANOVA followed by Bonferroni post hoc test). **C** Alkaline phosphatase staining of human VICs under osteogenic stimulus for 0 (i), 1 (ii), 3 (iii), 5 (iv) and 7 (v) days. Scale bar = 200 µm. **D** Quantifications of data presented in C (one-way ANOVA followed by Bonferroni post hoc test). **E** Western blotting and (**F**) quantitation for Rho A, ROCK1, MYPT1, p-MYPT1, OPN, RUNX2, GLUT1, LDHA, HK2, PFK1 and PDK1 of human VICs under osteogenic stimulus for 0, 1, 3, 5 and 7 days (one-way ANOVA followed by Bonferroni post hoc test). β-actin was used as loading control. Data are shown as mean ± SD. *P < 0.05, **P < 0.01, ***P < 0.001, ****P < 0.0001.

As an approach to stimulate calcification of VICs, human VICs were cultured in IP-OM for up to 7 days to construct an inorganic phosphorus-osteogenic induction model (IP-OIM) in vitro. During this process, calcification became gradually more extensive. Human VICs at day 1 showed no detectable calcified deposition, as examined by a deficiency of alizarin red reactivity (Fig. 2A ii), and minimal alkaline phosphatase activity (Fig. 2C ii). By day 3 of osteogenic stimulation, human VICs were first noted with a few scattered calcific nodules with narrow positive areas of alizarin red (Fig. 2A iii) and alkaline phosphatase staining (Fig. 2C iii). Human VICs under calcifying conditions at day 5 showed obviously extensive calcification revealed as increased mineralization nodules, and was positive for alizarin red (Fig. 2A iv) and alkaline phosphatase staining (Fig. 2C iv). By day 7, nearly 90% of human aortic VICs were detected calcified nodules, indicating a further increase in mineralization (Fig. 2A v and 2 C v). In line with these results (Fig. 2A to D), western blotting verified an increase in the expressions of known osteogenic markers, including RUNX2 and OPN, in a time-dependent manner (Figs. 2E and 2F). Accordingly, protein expressions of Rho A and ROCK1, and MYPT1 phosphorylation increased during this calcification process (Figs. 2E and 2F).

### ROCK1 inhibition promotes RUNX2 ubiquitination and proteasomal degradation

**Fig. 3:**
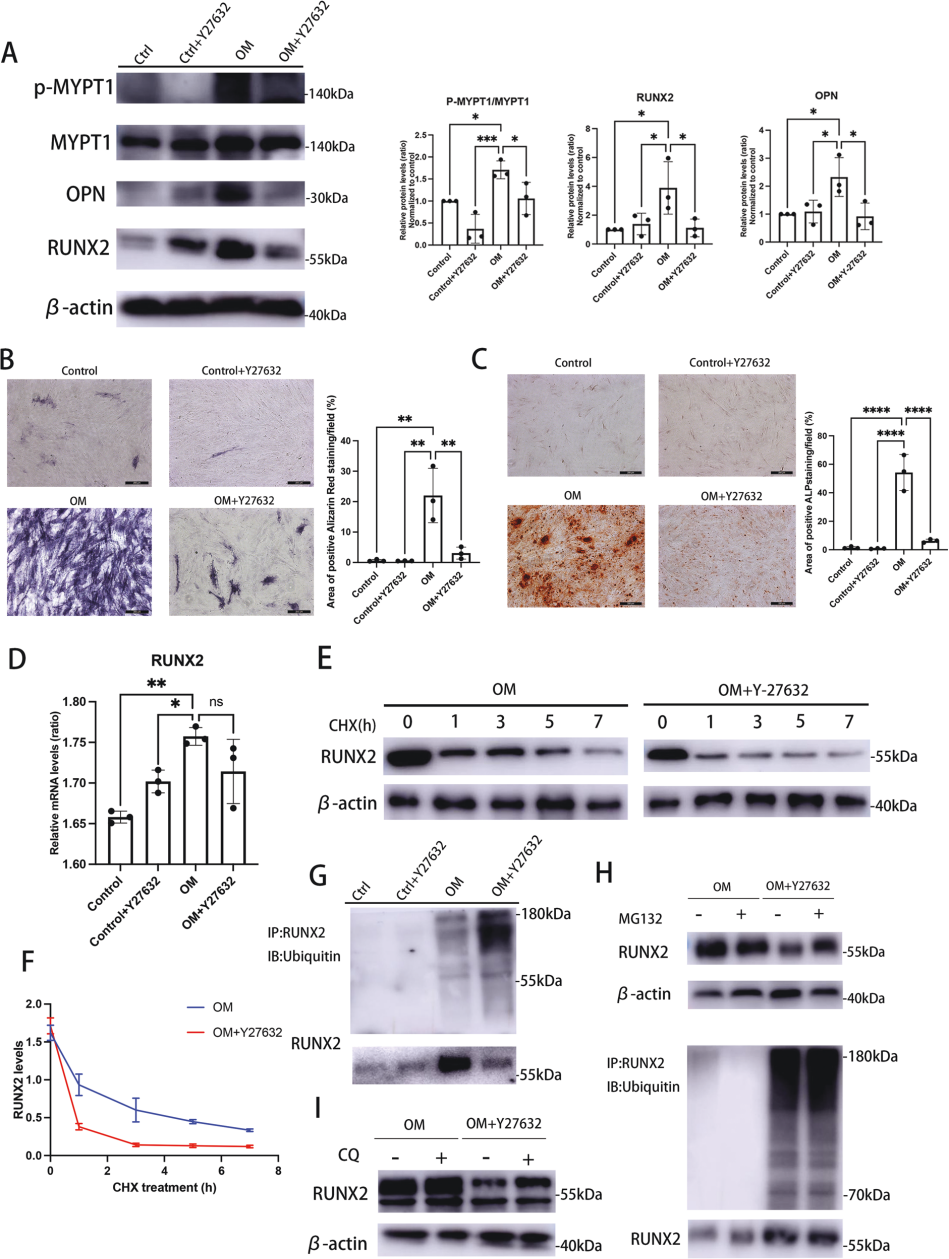
ROCK1 inhibition promotes RUNX2 ubiquitination and proteasomal degradation. **A** Western blot and quantification for p-MYPT1, MYPT1, OPN and RUNX2 after culturing human VICs in osteogenic medium (OM) or complete medium (CM) for 7 days in the presence or absence of Y27632 (150 nM) (one-way ANOVA followed by Bonferroni post hoc test). β-actin was used as loading control. **B** Representative images of ALP staining and quantitation, after culturing human VICs in OM or CM, in the presence of Y27632 (150 nM) or vehicle (DMSO) (one-way ANOVA followed by Bonferroni post hoc test). Scale bar = 200 µm. **C** Representative images of alizarin red staining and quantitation, after culturing human VICs in OM or CM, in the presence of Y27632 (150 nM) or vehicle (DMSO) (one-way ANOVA followed by Bonferroni post hoc test). Scale bar = 200 µm. **D** the mRNA levels of RUNX2 in human VICs after growth in CM or OM, in the presence or absence of Y27632 (150 nM) were determined using qRT-PCR (one-way ANOVA followed by Bonferroni post hoc test). **E** Lysates of OM-induced calcified human VICs with or without Y27632 (150 nM) treated with 50 ug/ml cycloheximide (CHX) for the indicated times were examined by western blot and (**F**) RUNX2 levels were quantified (one-way ANOVA followed by Bonferroni post hoc test). β-actin was used as loading control. **G** Ubiquitination of immune-precipitated RUNX2 in VICs after growth in CM or OM, in the presence or absence of Y27632 (150 nM). **H** Ubiquitination of immune-precipitated RUNX2, as well as RUNX2 protein levels of calcified VICs treated with or without Y27632 (150 nM), in the presence or absence of MG132 (20 μM). **I** VICs cultured in OM with or without Y27632 (150 nM) were treated in the presence or absence of CQ (20 μM), and examined for RUNX2 expression using immunoblotting. β-actin was used as loading control. Data are shown as mean ± SD. *P < 0.05, **P < 0.01, ***P < 0.001, ****P < 0.0001.

Treatment with Y-27632 (150 nM), a selective inhibitor of ROCK1, led to an apparent reduction in phosphorylation levels of MYPT1 and a significant alleviation of osteogenic changes of human VICs (Fig. 3A to C). Here, we noticed that ROCK1 inhibition decreased the endogenous RUNX2 protein abundance in calcified VICs from IP-OIM, but did not affect their mRNA levels (Fig. 3D), indicating that the regulation of Rho A/ROCK1 signaling on RUNX2 might occur mainly at its protein level. To determine whether the protein stability of RUNX2 was adjusted by Y-27632, we first blocked de novo protein synthesis through cycloheximide (CHX) and demonstrated that Y-27632 shortened the half-life of RUNX2 markedly in human calcifying VICs (Fig. 3E and F). Ubiquitin-proteasome pathway is a primary pathway mediating endogenous protein degradation in cells, and the degradation of RUNX2 protein mediated by ubiquitination shows essential effect on regulatory processes of calcification in mice osteoblasts [39]. Interestingly, immunoprecipitation showed that RUNX2 ubiquitination was markedly increased upon human VICs from IP-OIM that cultured in the presence of Y-27632, as compared to those calcific VICs cultured without Y-27632 (Fig. 3G). Furthermore, we noticed that RUNX2 accumulation was restored when Y27632-cultured calcifying VICs were treated with a proteasomal inhibitor MG132 and that RUNX2 ubiquitination level was concomitantly increased (Fig. 3H), whereas the lysosomal inhibitor chloroquine (CQ) exhibited no such effect on RUNX2 protein abundance (Fig. 3I). Taken together, these results validated Rho A/ROCK1 signaling modulates RUNX2 protein expressions in a ubiquitin-proteasome dependent manner.

### AMPK inhibition partially abolished the enhancing effect of Y-27632 on RUNX2 proteasome degradation

**Fig. 4:**
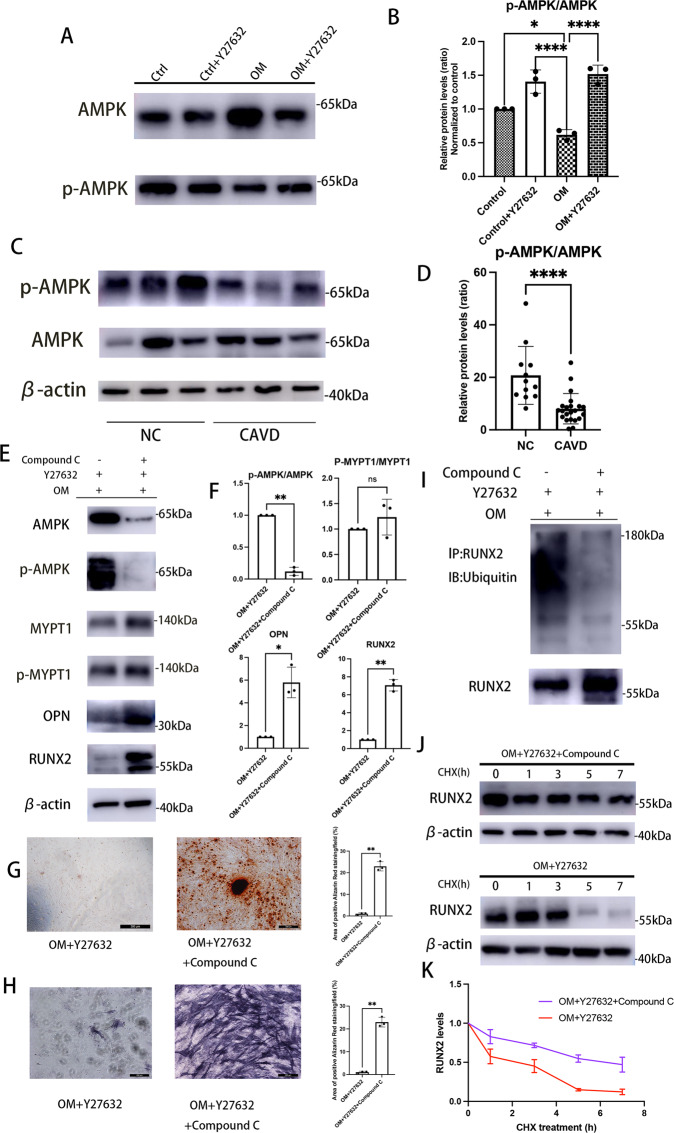
AMPK inhibition abolished Y-27632-enhanced RUNX2 proteasome degradation. **A** Western blot and quantification (**B**) for AMPK and p-AMPK in human VICs in osteogenic medium (OM) or complete medium (CM) treated with or without Y27632 (one-way ANOVA followed by Bonferroni post hoc test). **C** Western blotting for AMPK and p-AMPK of representative human aortic valves, either calcified or non-calcified. β-actin was used as loading control. **D** Quantification of AMPK and p-AMPK of human calcified (*n* = 22) and non-calcified aortic valves (*n* = 12) (Mann–Whitney test). **E** Western blotting for AMPK, p-AMPK, MYPT1, p-MYPT1, OPN and RUNX2 of OM-Y27632-cultured human VICs treated with or without Compound C. β-actin was used as loading control. **F** Quantifications of data presented in E (Unpaired two-tailed Student’s *t*-test). **G**, **H** Representative images and quantifications of alizarin red and ALP staining for OM-Y27632-cultured human VICs treated with or without Compound C. (unpaired two-tailed Student’s *t*-test). Scale bar = 200 µm. **I** Ubiquitination of immune-precipitated RUNX2 in OM-Y27632-cultured VICs treated in the presence or absence of Compound C. **J** Lysates of OM-Y27632-treated human VICs with or without Compound C treated with 50 ug/ml cycloheximide (CHX) for the indicated times were examined by western blot and (**K**) RUNX2 levels were quantified (one-way ANOVA followed by Bonferroni post hoc test). Data are shown as mean ± SD. *P < 0.05, **P < 0.01, ***P < 0.001, ****P < 0.0001.

In the course of these experiments, AMPK activity, which was assessed by the phosphorylation of its α subunit at Thr172 was found to be downregulated in human calcified VICs (Fig. 4A and B). Similarly, western blotting of human aortic valves also investigated a much lower phosphorylation level of AMPK in CAVs (Fig. 4C and D). However, phosphorylation of AMPK in calcifying human VICs could be upregulated to control levels after Y-27632 treatment (Fig. 4A and B). Previous studies have demonstrated that RUNX2 ubiquitination in mice osteoblasts is positively regulated by AMPK [39]. To demonstrate if the positive effect of Y-27632 on RUNX2 ubiquitin-proteasome degradation and subsequent repressive effect on VICs calcification was caused by an increase in AMPK activity, we, thereafter, blocked AMPK activity in Y27632-treated calcifying VICs using Compound C (120 nM), an AMPK inhibitor. Notably, the inhibitory effect of Y-27632 on RUNX2 accumulation and subsequent VICs calcification was largely blocked by Compound C, appearing as upregulated protein expressions of OPN and RUNX2 (Fig. 4E and F), also higher alizarin red reactivity (Fig. 4G) and ALP activity (Fig. 4H). Consistently, downregulation of AMPK activity dramatically decreased Y27632-induced upregulation of RUNX2 ubiquitination in calcified human VICs (Fig. 4I). Also, the half-life of RUNX2 of Y27632-treated calcifying VICs under conditions of Compound C were much higher than those only cultured with Y27632 (Fig. 4J and K). While no impact of AMPK inhibitor was observed on ROCK1 phosphorylation (Fig. 4E and F), indicating that AMPK mediates Y27632-induced reduction of VICs calcification through enhancing RUNX2 ubiquitin-proteasome degradation.

### Human VICs from IP-OIM and CAVs appeared Warburg effect as their metabolic phenotype

**Fig. 5:**
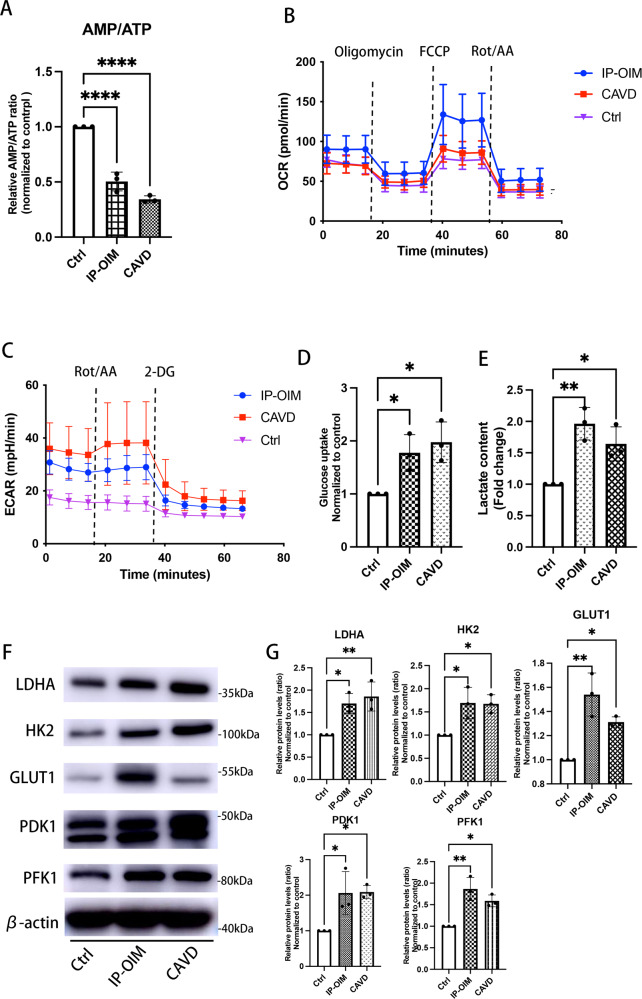
Human VICs from IP-OIM and CAVs appeared Warburg effect. **A** AMP/ATP ratio in human VICs isolated from non-calcified and calcified aortic valves, and human VICs from IP-OIM (one-way ANOVA followed by Bonferroni post hoc test). **B**, **C** Seahorse profiles for oxygen consumption rate (OCR) and extracellular acidification rate (ECAR) for human VICs isolated from non-calcified and calcified aortic valves, and human VICs from IP-OIM. **D**, **E** Glucose consumption rate and lactate content in calcified VICs from CAVs and IP-OIM, and normal VICs (one-way ANOVA followed by Bonferroni post hoc test). **F** Western blot and (**G**) quantification for LDHA, HK2, GLUT1, PDK1 and PFK1 in calcified VICs from CAVs and IP-OIM, and VICs isolated from non-CAVs (one-way ANOVA followed by Bonferroni post hoc test). β-actin was used as loading control. Data are shown as mean ± SD. *P < 0.05, **P < 0.01, ***P < 0.001, ****P < 0.0001.

AMPK activity was suppressed in calcifying VICs; therefore, we hypothesized that there would be metabolic reprogramming and a subsequent imbalance in adenosine content. We measured ATP, ADP and AMP contents in calcified human VICs. There were approximately 3- and 2-fold increase in ATP in human VICs under CAVD pathological conditions or IP-OM stimulation, leading to a 3- and 2-fold decrease in the ratio of AMP/ATP, respectively (Fig. 5A), which might contribute to the consequent suppression of the primary energy sensor, AMPK. Since Ca/P-induced calcifying VICs, as well as VSMCs and pre-osteoblasts generate almost of their ATP through glycolysis during osteogenic process, we wondered whether osteogenic VICs from IP-OIM and CAVs also behaved Warburg effect as their characteristic features. Surprisingly, both human VICs from CAVs and IP-OIM showed stronger capacity of glycolysis, but not OXPHOS, indicative of metabolic preference to Warburg effect (Fig. 5B and C). IP-induced osteogenic VICs took up approximately 1.5-fold quantity of 2-DG taken up by normal VICs, while a similar tendency was showed in VICs isolated from human CAVs (Fig. 5D). Moreover, calcifying VICs induced a substantially increase in lactic acids accumulation than controls as examined by colorimetric methods (Fig. 5E). Along, upregulation of metabolism-related proteins required for glycolysis, including HK2, PFK1, PDK1, LDHA and GLUT1 were detected in human VICs both undergoing CAVD pathological stimulation in vivo and accepting IP-OM stimulation in vitro (Fig. 5F and G).

### Key transporters and enzymes for Warburg effect were upregulated in human calcified aortic valves

**Fig. 6:**
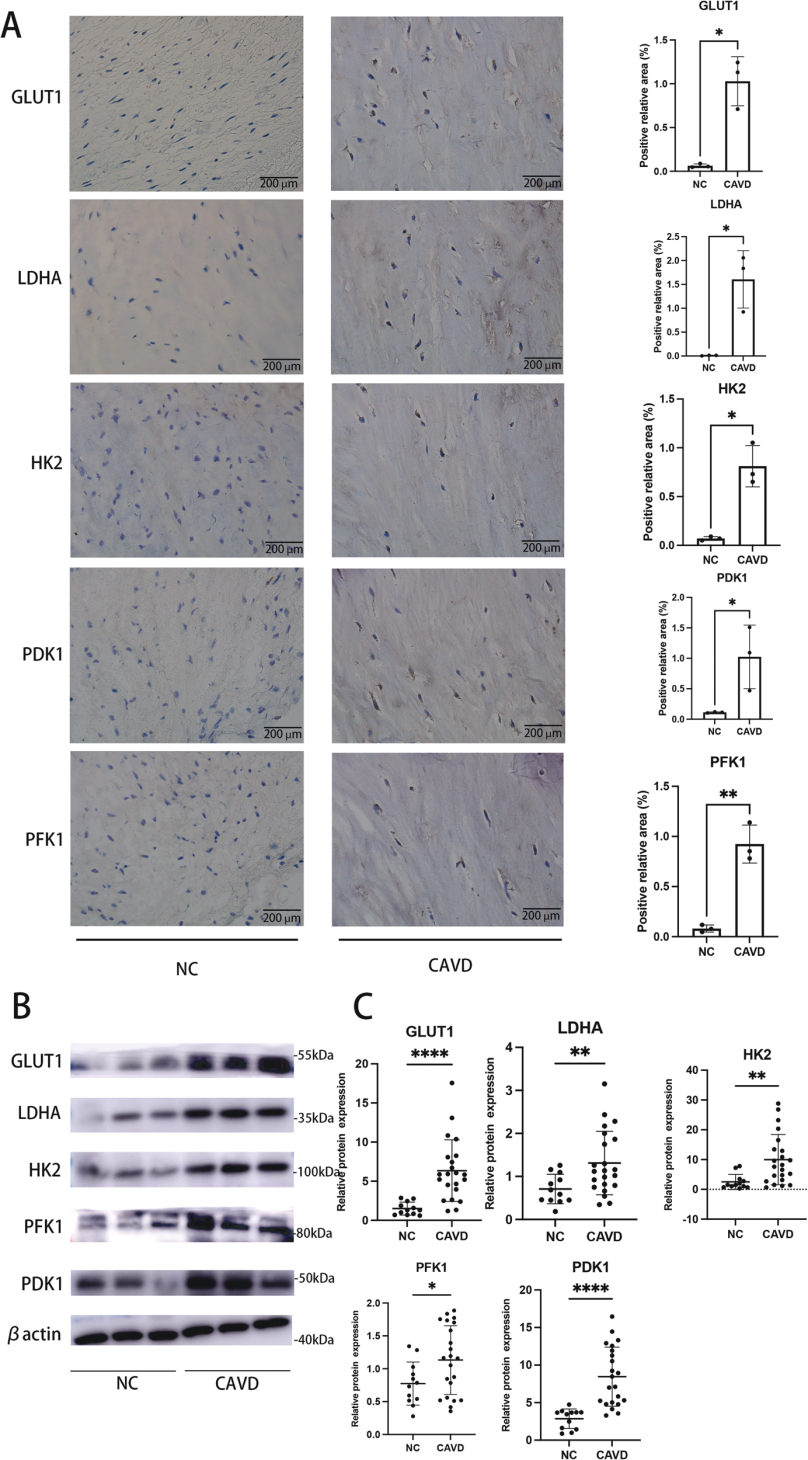
Warburg-associated key proteins are upregulated in human calcified aortic valves. **A** Representative immunohistochemical images, and quantifications for % GLUT1^+^, % LDHA^+^, % HK2^+^, % PFK1^+^ and % PDK1^+^ area in human aortic valves with and without CAVD (Unpaired two-tailed Student’s t-test with Welch’s correction for GLUT1, HK2 and LDHA; unpaired two-tailed Student’s t-test for PDK1 and PFK1). Scale bar = 200 µm. **B** Representative images and (**C**) quantifications of western blot of GLUT1, LDHA, HK2, PFK1 and PDK1 in human calcified aortic valves (*n* = 22) versus control (*n* = 12) (Unpaired two-tailed Student’s *t*-test for GLUT1 and PFK1; Mann–Whitney test for HK2; unpaired two-tailed Student’s *t*-test with Welch’s correction for LDHA and PDK1). β-actin was used as loading control. Data are shown as mean ± SD. *P < 0.05, **P < 0.01, ***P < 0.001, ****P < 0.0001.

Based on the metabolic reprogramming toward the Warburg effect in osteogenic VICs we previously confirmed, herein, we further indirectly assessed whether Warburg effect exists in human CAVD process in vivo. Locally, immunohistochemistry analysis confirmed abundant GLUT1, HK2, PDK1, PFK1 and LDHA levels in calcified regions of aortic valves leaflets (Fig. 6A), correlated with upregulation of Rho A/ROCK1 signaling (Fig. 1A to D), but were barely detectable in the non-calcified valve samples (Fig. 6A). Similarly, samples from patients with CAVD presented higher expressions of GLUT1, HK2, PDK1, PFK1 and LDHA at protein levels, versus controls (Figs. 6B and 6C). Changes in the rate of glycolysis could not be observed using valve tissues, but the increased expressions of key transporters and enzymes associated with Warburg effect reinforced the idea that aerobic glycolysis occurred in human model of CAVD in vivo.

### Inhibition of Warburg effect leads to human VICs calcification loss

**Fig. 7:**
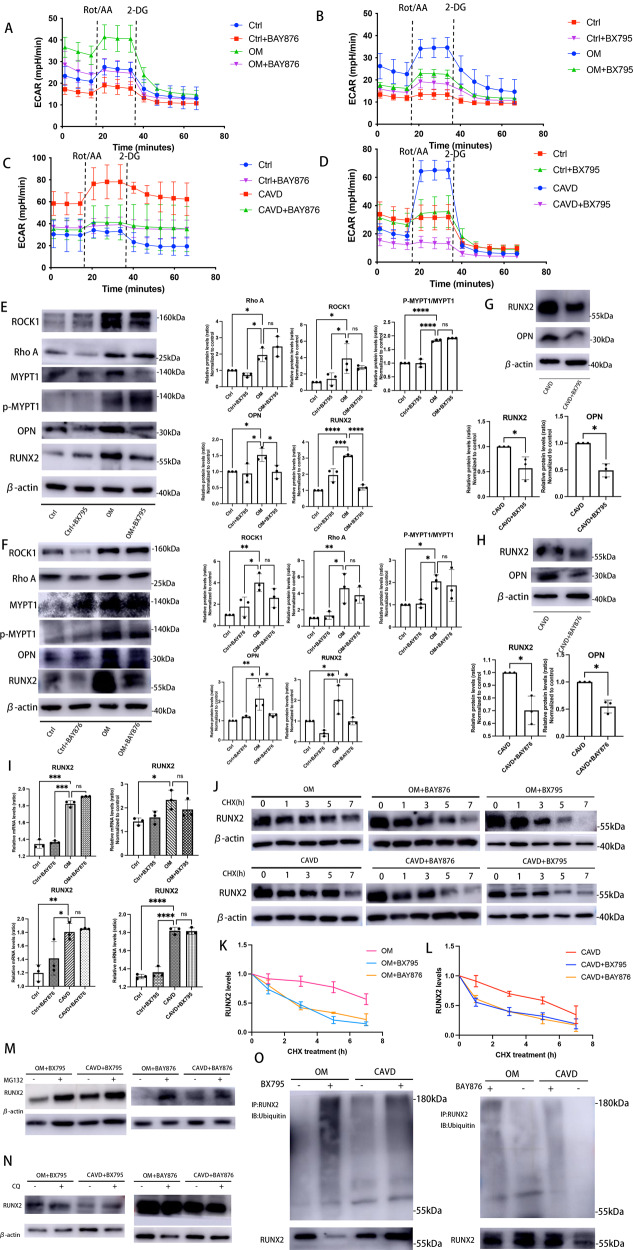
Inhibition of Warburg effect leads to human VICs calcification loss. **A**–**D** Seahorse profiles for extracellular acidification rate (ECAR) of calcified VICs from CAVs or IP-OIM, and non-calcified VICs isolated from non-CAVs, cultured in the presence or absence of BAY876/BX795. **E**, **F** Western blot and quantification for ROCK1, Rho A, MYPT1, p-MYPT1, OPN and RUNX2 in human VICs cultured in OM or CM treated with BX795/BAY876 (one-way ANOVA followed by Bonferroni post hoc test). β-actin was used as loading control. **G**, **H** Western blot and quantification for OPN and RUNX2 in human VICs isolated from CAVs treated with BAY876/BX795 (unpaired two-tailed Student’s *t*-test with Welch’s correction). β-actin was used as loading control. **I** the mRNA levels of RUNX2 in human VICs from IP-OIM or CAVs, cultured in the presence or absence of BAY876/BX795, were determined using qRT-PCR (one-way ANOVA followed by Bonferroni post hoc test). **J** Lysates of calcified human VICs from IP-OIM and CAVs with or without BAY876/BX795 treated with 50 ug/ml cycloheximide (CHX) for the indicated times were examined by western blot and (**K**, **L**) RUNX2 levels were quantified (one-way ANOVA followed by Bonferroni post hoc test). β-actin was used as loading control. **M** RUNX2 protein levels of calcified VICs from IP-OIM or CAVs treated with or without BAY876/BX795, in the presence or absence of MG132 (20 μM). β-actin was used as loading control. **N** VICs from IP-OIM or CAVs with or without BAY876/BX795 were treated in the presence or absence of CQ (20 μM), and examined for RUNX2 expression using immunoblotting. β-actin was used as loading control. **O** Ubiquitination of immune-precipitated RUNX2 in VICs from IP-OIM or CAVs, in the presence or absence of BAY876/BX795. Data are shown as mean ± SD. *P < 0.05, **P < 0.01, ***P < 0.001, ****P < 0.0001.

Aerobic glycolysis displayed a positive association with human VICs calcification as our data confirmed. Of note, our observations showed HK2, PFK1 and LDHA proteins in IP-OIM were not upregulated in a time-dependent manner, and an expression peak of HK2 and LDHA was found in advanced of osteogenic markers (Fig. 2E and F), indicating metabolism switching in VICs maybe ahead of its calcification. However, it is still unclear whether metabolic effects regulate human VICs osteogenic differentiation directly or are evident only when VICs osteogenic changes established. We used highly selective GLUT1 inhibitor, BAY-876 (2 nM) and specific PDK1 inhibitor, BX-795 (6 nM), to decrease glycolysis in calcifying VICs. Both PDK1 and GLUT1 inhibition reduced glycolysis in OM-induced calcified VICs (Fig. 7A and B) as well as VICs isolated from human aortic valves under calcific pathological conditions (CAVD) (Fig. 7C and D). The glycolytic flux in calcifying VICs cultured with BAY-876 or BX-795 was abrogated to normal levels, and consequently, osteogenic markers, RUNX2 and OPN (Fig. 7E to H), were markedly reduced. Also, no significant changes were found in *RUNX2* mRNA expressions after GLUT1 or PDK1 inhibition treatments (Fig. 7I). Furthermore, we found that the half-life of RUNX2 apparently decreased after GLUT1 or PDK1 inhibition (Fig. 7J to L). The inhibitory effect of BAY876 and BX795 on the RUNX2 protein expressions were significantly reversed by MG132 (Fig. 7M), but not CQ (Fig. 7N). Motivated by above results, we therefore performed immunoprecipitation experiments and found that RUNX2 ubiquitination levels in BAY876-treated and BX795-treated VICs were much higher than those in calcified VICs treated without BAY876 or BX795 (Fig. 7O).

### Rho A/ROCK1 signaling positively regulate Warburg effect in osteogenic human VICs

**Fig. 8:**
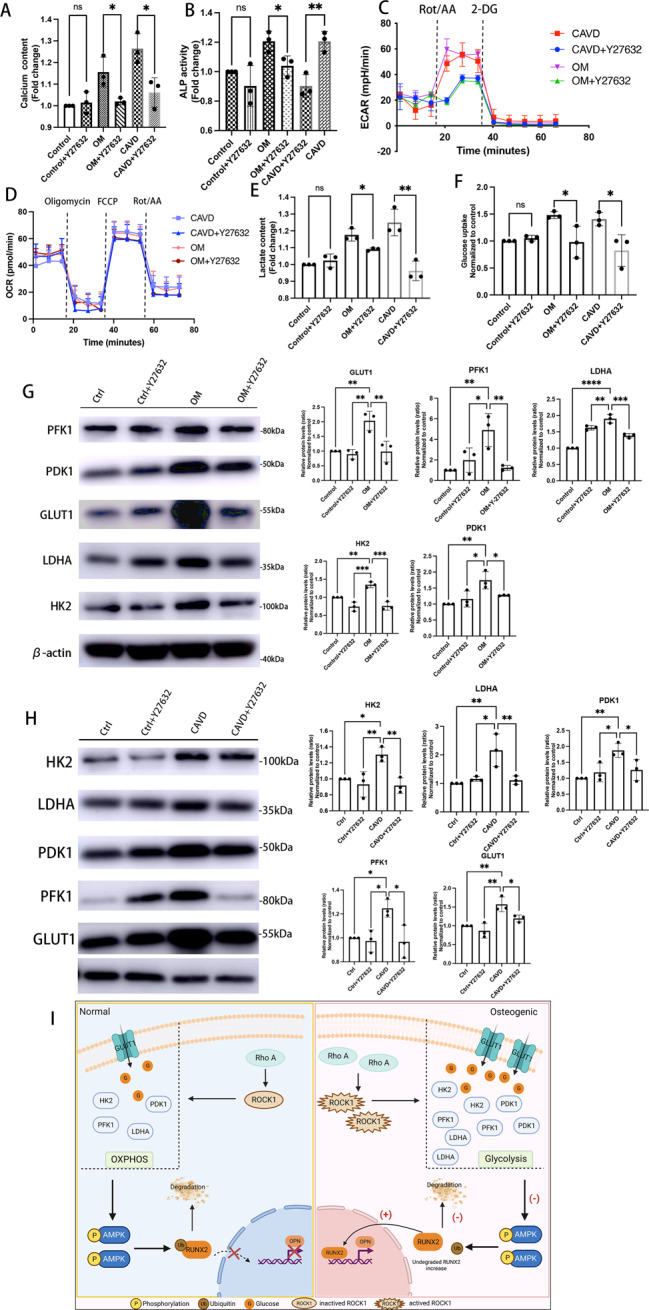
Rho A/ROCK1 signaling positively regulate Warburg effect in osteogenic human VICs. **A**, **B** Calcium content and ALP activity was quantified in human aortic VICs from IP-OIM, non-CAVs and CAVs, treated with or without Y27632 (Unpaired two-tailed Student’s *t*-test). **C**, **D** Seahorse profiles for extracellular acidification rate (ECAR) of VICs from IP-OIM, non-CAVs and CAVs, cultured in the presence or absence of Y27632. **E**, **F** Lactate content and glucose consumption rate in human VICs isolated from IP-OIM, non-CAVs and CAVs, cultured with or without Y27632 (Unpaired two-tailed Student’s *t*-test). **G**, **H**, Western blot analysis and quantification for GLUT1, LDHA, HK2, PFK1 and PDK1 protein expression in human aortic VICs from IP-OIM, non-CAVs and CAVs, cultured in the presence or absence of Y27632. (one-way ANOVA followed by Bonferroni post hoc test). β-actin was used as loading control. **I** A graphical abstract (painted by Biorender). Elevated Rho A/ROCK1 signaling drives metabolic switching of VICs to the Warburg effect, suppresses AMPK activity and subsequently attenuates RUNX2 ubiquitin-proteasome degradation, leading to increased RUNX2 protein accumulation and thus osteogenic differentiation of VICs. Data are shown as mean ± SD. *P < 0.05, **P < 0.01, ***P < 0.001, ****P < 0.0001.

Against this background, we continued to analyze whether Warburg effect in calcified human VICs was regulated by Rho A/ROCK1 signaling. Treatment with BAY-876 or BX-795 markedly reduced glycolysis in human VICs while did not affect neither protein expression of Rho A or ROCK1, nor ROCK1 phosphorylation (Fig. 7E and F). Calcium deposits (Fig. 8A) and ALP activity (Fig. 8B) were intensely negated in VICs treated with Y-27632. As shown in Figs. 8C and 8D, both human VICs from the IP-OIM and CAVs groups behaved decreased ECAR when treated with Y-27632, while seahorse analysis did not show altered OCR, representing the successful inhibition of the Warburg effect by Y-27632 (Fig. 8C and D). Analysis of lactic acid levels using colorimetric methods revealed significantly lower lactate concentrations after treatment with Y-27632, compared with calcifying groups (Fig. 8E). Consistently, the decrease in glycolysis was accompanied by marked reduction in the protein expressions of GLUT1, HK2, PFK1, PDK1 and LDHA (Fig. 8G and H). In addition, calcified VICs cultured in combination with Y-27632 exhibited reduced 2-DG fluorescence compared with those cultured without Y-27632 (Fig. 8F). Collectively, these results suggested that Rho A/ROCK1 blocking partially reversed the metabolic reprogramming toward the Warburg effect in human calcifying VICs.

## Discussion

Complicated and volatile hemodynamic environment as the aortic valves residing in, abnormal mechanical strains is thought to be a crucial event in the pathogenesis of CAVD, which was reinforced by enhanced VICs mineralization in disrupted extracellular matrix (ECM) homeostasis [40], as well as accelerated CAVD progression in patients with bicuspid aortic valves [41–43]. Rho A/ROCK signaling was identified as a side- and shear-dependent pathway [44], sensing potentially destructive hemodynamic forces and transducing mechanical signals into cellular responses [45, 46]. Although the positive association between Rho A/ROCK and CAVD has been confirmed, the cellular mechanisms involved are still poorly understood. VICs, the major cellular component of aortic valves, play a positive role in triggering calcification as its osteogenic transition potency [10, 47]. In this study, we first demonstrated that Rho A/ROCK1 signaling promotes aortic valves calcification via upregulating a novel metabolic characteristic of human VICs. Specifically, we observed that Rho A/ROCK1, but not ROCK2, signaling was significantly upregulated in human calcified aortic valves, human VICs isolated from CAVs, and human VICs undergoing OM-induced calcification. Rho A/ROCK1 signaling aggravated human VICs calcification via upregulating VICs’ metabolic reprogramming to Warburg effect, which subsequently reduced AMPK activity. AMPK inhibition reduced RUNX2 ubiquitin-proteasome degradation, thus promoted RUNX2 accumulation and a final calcification of VICs (Fig. 8I).

Aberrant upregulation of RUNX2, a necessary osteogenic transcription factor associated with osteogenesis, has been confirmed as an essential initiator for noxious cellular events that drive calcified pathology in cardiovascular system [48]. Mounting evidence indicates that ubiquitin-mediated proteasomal degradation of RUNX2 and subsequent modification of RUNX2 protein levels play pivotal roles in multiple pathogenesis of calcification procedures, such as, skeletal development [39] and atherosclerosis [49, 50]. Herein, we confirmed that ubiquitination of RUNX2 was reduced in calcified human VICs, resulting in an increase in RUNX2 accumulation and associated enhancement of osteogenic changes. This was consistent with previous findings, indicating that ubiquitination might also act as a crucial posttranslational modulator for RUNX2 protein fate by signing subunits for proteasomal degradation during CAVD progression [51].

Previous studies reported that applying IP-OIM or 3-D hydrogels with stress strain to create osteogenic conditions for VICs in vitro showed increased Rho A/ROCK1 signaling, while inhibition of Rho A concurrent with osteogenic treatment reduced calcific nodule formation [40, 52]. Moreover, the Nfatc1^Cre^; R26-Cad11^Tg/Tg^ mouse model developed hemodynamically significant aortic stenosis with prominent calcific lesions, which also correlated with upregulation of Rho A in aortic valve leaflets [28]. These previous findings indicated a positive role of Rho A/ROCK1 signaling in the CAVD process. However, its role in human CAVD remains poorly elucidated. Here, we detected for the first time that Rho A/ROCK1 signaling was abundant in human CAVs and in calcified VICs isolated from human CAVs, which was essentially consistent with the same upregulation in human VICs undergoing OM-induced calcification in vitro. Of note, we observed significant suppression of osteogenic differentiation during ROCK1 inhibition, accompanied by marked upregulation of RUNX2 ubiquitin-proteasome degradation. By contrast, loss of ROCK1 activity had no effect on *RUNX2* mRNA levels. These results indicated that Rho A/ROCK1 signaling might exert a regulatory effect on RUXN2 accumulation through ubiquitin-proteasome system; therefore, we further explored the intermediate mechanism involved.

Increasing evidence suggests that AMPK-mediated signaling pathways regulate CAVD, arteriosclerosis [32, 53] and skeleton development [39]. More precisely, AMPK negatively regulated osteogenic differentiation, thereby restraining calcification and significantly decreasing RUNX2 protein expression when AMPK activity was upregulated. AMPK inhibition apparently contributed to RUNX2-dependent atherosclerotic calcification [32]. A recent study verified that metformin had potent effects on alleviating mineralization of human VICs via its activation of AMPK; however, whether the final level of RUNX2 was affected remained unreported [35]. AMPK inhibition is assuredly relevant to CAVD, which was verified in human CAVs, and human VICs from IP-OIM in our work, consistent with previous studies. We showed that ROCK1 activity inhibition not only reduced the calcification of human VICs, but significantly recovered AMPK phosphorylation. Accordingly, whether Rho A/ROCK1 regulates RUNX2 ubiquitin-proteasome degradation in human VICs in an AMPK-dependent manner or is merely an accompanying phenomenon was investigated. A recent work showed that intensive AMPK activity in osteoblasts was deleterious for bone formation, mainly through favoring RUNX2 proteasomal degradation via phosphorylating Smurf1, an E3 ubiquitin ligase tightly associated with ubiquitin-proteasome degradation. Similar to osteoblasts, we provided evidence that Y27632-mediated RUNX2 ubiquitination and proteasome degradation could be interrupted by AMPK inhibition. This variability reflected an AMPK-dependent regulatory mechanism involved in the ubiquitin-associated RUNX2 degradation during the activation of Rho A/ROCK1.

AMPK, functioning as an energy sensor and modulator, responds to variations of intracellular adenine contents and was activated by an increase in the ratio of AMP/ADP to ATP, ultimately regulating cellular energy homeostasis [54]. Recently, AMPK was found to participate in the pathogenesis of multiple metabolic disorders, such as type 2 diabetes, cancer, and lipotoxic cardiomyopathy [55]. Several osteogenic processes appeared lower AMPK activity, and this variability might result from specific metabolic reprogramming, which could make cells produce ATP more rapidly. Indeed, studies showed that AMPK activity was negatively regulated by aerobic glycolysis in cancer cells, while deletion of the α1 catalytic subunit of AMPK accelerated lymphomagenesis [56, 57]. A recent report showed that calcium/phosphorus (Ca/P)-induced calcified human VICs displayed an energy switch in adenosine triphosphate (ATP) production from mitochondrial OXPHOS to aerobic glycolysis [24]. Similar to Ca/P-induced calcification, calcified human VICs from IP-OIM and CAVs acquired glycolytic states, which promote CAVD via their metabolism-related pathways. Consistent with these findings, we verified for the first time that key enzymes and transporters demanded for glycolysis were highly expressed in human CAVs, human VICs isolated from CAVs, and human VICs undergoing IP-OM stimulation. Known as nucleated cells, VICs have two optional manners to generate ATP. While the ATP production per molecule of glucose is much lower for aerobic glycolysis compared to OXPHOS, the metabolic transition to Warburg effect allows cells to yield energy more rapidly, which confer both bioenergetic and biosynthetic advantages on differentiating cells [56, 58]. Thus, it was not surprising that VICs displayed Warburg effect as its characteristic features. Furthermore, vascular calcification is a pathological process similar to valve calcification, in which the osteogenic differentiation of VSMCs serves as its key point [59]. During vascular injury, VSMCs switch from a quiescent phenotype to a highly proliferative phenotype [60]. Intimal smooth muscle cells from plaques of rabbit carotid artery exhibited an increased capacity of glycolysis [61]. Moreover, studies have reported aberrant upregulation of key glycolytic enzymes in calcified vessels [60, 62–64]. While attenuating atherosclerosis through depriving glycolysis of VMSCs further proved the causality between osteogenic changes and glycolytic preference [62]. Additionally, prospective osteoblasts also displayed metabolic switching towards glycolysis [39], together with a shift in AMPK activity, and the normal expression of *RUNX2* cannot induce calcification when glucose uptake was hampered. Clinical studies demonstrated that some patients with cleidocranial dysplasia (CCD) showed no detectable mutations in *RUNX2*, while *GLUT1*^−/−^ embryos developed CCD, further indicating that CCD might be caused by a decrease in glucose consumption [65]. These data emphasized the importance of metabolic reprogramming toward glycolysis in the progression of calcification. Therefore, our findings provided new mechanistic insights into the concept of CAVD, suggesting that it might be appropriate to list CAVD as a metabolic disease.

In the present study, calcifying human VICs showed a significant increase in ECAR, but not OCR. These variations were in parallel with Warburg effect, which occurs in the metabolic signature of 70–80% of human cancers, strongly associated with the invasive behavior of tumor cells. ROCK1 has the ability to drive cortical actomyosin contraction and is closely related to tumor metastasis [66–69]. Inhibition of Rho A/ROCK1 signaling was found to abolish the function of tumor-associated mutant p53 (mutp53) proteins by inhibiting GLUT1 translocation to the plasma membrane, consequently leading to an inhibition of glycolysis and tumorigenesis [21]. In addition, it was also reported that ROCK1 could regulate the Warburg effect and accelerated ovarian cancer growth [70]. However, it was unclear whether the essential metabolic modifications could be detected in human VICs during osteogenic progression. Here, we showed that ROCK1 inhibition dramatically decreased the Warburg effect. These data reflect a regulatory function of Rho A/ROCK1 signaling for the Warburg effect in human VICs, indicating a link between mechanical strain and cellular metabolism.

In conclusion, our findings demonstrated that Rho A/ROCK1 signaling positively regulates RUNX2 accumulation via a reduction of AMPK-dependent ubiquitin-associated RUNX2 proteasomal degradation caused by an enhanced Warburg effect during osteogenic differentiation of VICs. Mechanistically, our study provides novel insights into the molecular mechanism for Rho A/ROCK1 signaling in controlling CAVD, highlighting metabolic disorders in the pathogenesis of CAVD, and emphasizing a new button between abnormal hemodynamics and the metabolic phenotype.

## Supplementary information

Extended Figure S1

Supplementary figure legends

Supplemental Table S2

original data files

checklist

## Data Availability

The authors declare that the majority of supporting data are presented within this article or in Data Supplement. Data not directly available that support the findings of this study are available from the corresponding author upon reasonable request.

## References

[1] GBD 2017 Causes of Death Collaborators. Global, regional, and national age-sex-specific mortality for 282 causes of death in 195 countries and territories, 1980-2017: a systematic analysis for the Global Burden of Disease Study 2017. Lancet (London, England). 2018;392:1736-88. 10.1016/S0140-6736(18)32203-7 PMC6227606 30496103

[2] Lindman BR, Clavel MA, Mathieu P, Iung B, Lancellotti P, Otto CM (2016). Calcific aortic stenosis. Nat Rev Dis Prim.

[3] Zheng K, Tzolos E, Dweck M (2020). Pathophysiology of aortic stenosis and future perspectives for medical therapy. Cardiol Clin.

[4] Li N, Bai Y, Zhou G, Ma Y, Tan M, Qiao F (2020). miR-214 attenuates aortic valve calcification by regulating osteogenic differentiation of valvular interstitial cells. Mol Ther Nucleic acids.

[5] Baumgartner H, Falk V, Bax J, De Bonis M, Hamm C, Holm P, et al. ESC/EACTS guidelines for the management of valvular heart disease. Rev Espanola De Cardiologia (Engl ed). 2018;71:110. 10.1016/j.rec.2017.12.013 29425605

[6] Lee Y, Chou Y (1998). Pathogenetic mechanism of senile calcific aortic stenosis: the role of apoptosis. Chin Med J.

[7] Mohler E, Gannon F, Reynolds C, Zimmerman R, Keane M, Kaplan F (2001). Bone formation and inflammation in cardiac valves. Circulation.

[8] Miller JD, Chu Y, Brooks RM, Richenbacher WE, Peña-Silva R, Heistad DD (2008). Dysregulation of antioxidant mechanisms contributes to increased oxidative stress in calcific aortic valvular stenosis in humans. J Am Coll Cardiol.

[9] Boström KI, Rajamannan NM, Towler DA (2011). The regulation of valvular and vascular sclerosis by osteogenic morphogens. Circ Res.

[10] Gould S, Srigunapalan S, Simmons C, Anseth K (2013). Hemodynamic and cellular response feedback in calcific aortic valve disease. Circ Res.

[11] Aguado B, Walker C, Grim J, Schroeder M, Batan D, Vogt B (2022). Genes that escape X chromosome inactivation modulate sex differences in valve myofibroblasts. Circulation.

[12] Morvan M, Arangalage D, Franck G, Perez F, Cattan-Levy L, Codogno I (2019). Relationship of iron deposition to calcium deposition in human aortic valve leaflets. J Am Coll Cardiol.

[13] Lampis A, Hahne J, Gasparini P, Cascione L, Hedayat S, Vlachogiannis G (2021). MIR21-induced loss of junctional adhesion molecule A promotes activation of oncogenic pathways, progression and metastasis in colorectal cancer. Cell death Differ.

[14] Barney L, Hall C, Schwartz A, Parks A, Sparages C, Galarza S (2020). Tumor cell-organized fibronectin maintenance of a dormant breast cancer population. Sci Adv.

[15] Singh S, Ray L, Shahi Thakuri P, Tran S, Konopka M, Luker G (2020). Organotypic breast tumor model elucidates dynamic remodeling of tumor microenvironment. Biomaterials.

[16] Daniel S, Seo Y, Pillarisetty V (2020). The CXCL12-CXCR4/CXCR7 axis as a mechanism of immune resistance in gastrointestinal malignancies. Semin cancer Biol.

[17] Tang Y, He Y, Zhang P, Wang J, Fan C, Yang L (2018). LncRNAs regulate the cytoskeleton and related Rho/ROCK signaling in cancer metastasis. Mol cancer.

[18] Seki T, Carroll F, Illingworth S, Green N, Cawood R, Bachtarzi H (2011). Tumour necrosis factor-alpha increases extravasation of virus particles into tumour tissue by activating the Rho A/Rho kinase pathway. J Control Release: Off J Control Release Soc.

[19] Seshacharyulu P, Rachagani S, Muniyan S, Siddiqui J, Cruz E, Sharma S (2019). FDPS cooperates with PTEN loss to promote prostate cancer progression through modulation of small GTPases/AKT axis. Oncogene.

[20] Mah EJ, Lefebvre A, McGahey GE, Yee AF, Digman MA (2018). Collagen density modulates triple-negative breast cancer cell metabolism through adhesion-mediated contractility. Sci Rep.

[21] Zhang C, Liu J, Liang Y, Wu R, Zhao Y, Hong X (2013). Tumour-associated mutant p53 drives the Warburg effect. Nat Commun.

[22] Fedele M, Sgarra R, Battista S, Cerchia L, Manfioletti G (2022). The epithelial-mesenchymal transition at the crossroads between metabolism and tumor progression. Int J Mol Sci.

[23] Chen C, Lin C, Kung H (2021). Targeting mitochondrial OXPHOS and their regulatory signals in prostate cancers. Int J Mol Sci.

[24] Wang S, Yu H, Gao J, Chen J, He P, Zhong H (2022). PALMD regulates aortic valve calcification via altered glycolysis and NF-κB-mediated inflammation.. J Biol Chem.

[25] Lerman DA, Prasad S, Alotti N (2016). Using Na(3)PO(4) to enhance in vitro animal models of aortic valve calcification. Int J Cardiovasc Res.

[26] Gu X, Masters KS (2011). Role of the Rho pathway in regulating valvular interstitial cell phenotype and nodule formation. Am J Physiol Heart Circ Physiol.

[27] Bouchareb R, Boulanger MC, Fournier D, Pibarot P, Messaddeq Y, Mathieu P (2013). Abstract 13341: Spheroid mineralized microparticles in calcific aortic stenosis are produced by valve interstitial cells: implication of mechanical strain and rhoa. Circulation.

[28] Sung DC, Bowen CJ, Vaidya KA, Zhou J, Chapurin N, Recknagel A (2016). Cadherin-11 overexpression induces extracellular matrix remodeling and calcification in mature aortic valves. Arterioscler Thromb Vasc Biol.

[29] Bowen CJ, Zhou J, Sung DC, Butcher JT (2015). Cadherin-11 coordinates cellular migration and extracellular matrix remodeling during aortic valve maturation. Dev Biol.

[30] Lu C, Dong X, Yu WP, Ding JL, Yang W, Gong Y (2020). Inorganic phosphate-osteogenic induction medium promotes osteogenic differentiation of valvular interstitial cells via the BMP-2/Smad1/5/9 and RhoA/ROCK-1 signaling pathways. Am J Transl Res.

[31] Day E, Ford R, Steinberg G (2017). AMPK as a therapeutic target for treating metabolic diseases. Trends Endocrinol Metab: TEM.

[32] Dai X, Liu S, Cheng L, Huang T, Guo H, Wang D (2022). Epigenetic upregulation of H19 and AMPK inhibition concurrently contribute to S-adenosylhomocysteine hydrolase deficiency-promoted atherosclerotic calcification. Circ Res.

[33] Singh A, Chaube B, Zhang X, Sun J, Citrin K, Canfrán-Duque A, (2021). Hepatocyte-specific suppression of ANGPTL4 improves obesity-associated diabetes and mitigates atherosclerosis in mice. J Clin Investig.

[34] Zhong S, Li L, Zhang Y, Zhang L, Lu J, Guo S (2019). Acetaldehyde dehydrogenase 2 interactions with LDLR and AMPK regulate foam cell formation. The. J Clin Investig.

[35] En Q, Zeping H, Yuetang W, Xu W, Wei W (2021). Metformin alleviates the calcification of aortic valve interstitial cells through activating the PI3K/AKT pathway in an AMPK dependent way. Mol Med (Camb, Mass).

[36] Meng X, Ao L, Song Y, Babu A, Yang X, Wang M (2008). Expression of functional toll-like receptors 2 and 4 in human aortic valve interstitial cells: potential roles in aortic valve inflammation and stenosis. Am J Physiol Cell Physiol.

[37] Majumdar U, Manivannan S, Basu M, Ueyama Y, Blaser MC, Cameron E (2021). Nitric oxide prevents aortic valve calcification by S-nitrosylation of USP9X to activate NOTCH signaling. Sci Adv.

[38] Yu C, Li L, Xie F, Guo S, Liu F, Dong N (2017). LncRNA TUG1 sponges miR-204-5p to promote osteoblast differentiation through upregulating Runx2 in aortic valve calcification. Cardiovasc Res.

[39] Wei J, Shimazu J, Makinistoglu M, Maurizi A, Kajimura D, Zong H (2015). Glucose uptake and Runx2 synergize to orchestrate osteoblast differentiation and bone formation. Cell.

[40] Duan B, Yin Z, Hockaday Kang L, Magin RL, Butcher JT (2016). Active tissue stiffness modulation controls valve interstitial cell phenotype and osteogenic potential in 3D culture. Acta Biomater.

[41] Shen M, Tastet L, Capoulade R, Arsenault M, Bédard É, Clavel M (2020). Effect of bicuspid aortic valve phenotype on progression of aortic stenosis. Eur Heart J Cardiovasc Imaging.

[42] Voisine M, Hervault M, Shen M, Boilard A, Filion B, Rosa M (2020). Age, sex, and valve phenotype differences in fibro-calcific remodeling of calcified aortic valve. J Am Heart Assoc.

[43] Shen M, Tastet L, Capoulade R, Larose É, Bédard É, Arsenault M (2017). Effect of age and aortic valve anatomy on calcification and haemodynamic severity of aortic stenosis. Heart (Br Card Soc).

[44] Hodge R, Ridley A (2016). Regulating Rho GTPases and their regulators. Nat Rev Mol cell Biol.

[45] Riou P, Villalonga P, Ridley A (2010). Rnd proteins: multifunctional regulators of the cytoskeleton and cell cycle progression. BioEssays: N Rev Mol Cell Dev Biol.

[46] Arimura N, Kaibuchi K (2005). Key regulators in neuronal polarity. Neuron.

[47] Butcher J, Simmons C, Warnock J (2008). Mechanobiology of the aortic heart valve. J heart Valve Dis.

[48] Chen Y, Zhao X, Wu H (2021). Transcriptional programming in arteriosclerotic disease: a multifaceted function of the runx2 (runt-related transcription factor 2). Arterioscler Thromb Vasc Biol.

[49] Wang Y, Han D, Zhou T, Chen C, Cao H, Zhang JZ (2021). DUSP26 induces aortic valve calcification by antagonizing MDM2-mediated ubiquitination of DPP4 in human valvular interstitial cells. Eur Heart J.

[50] Deng L, Huang L, Sun Y, Heath JM, Wu H, Chen Y (2015). Inhibition of FOXO1/3 promotes vascular calcification. Arterioscler Thromb Vasc Biol.

[51] Popovic D, Vucic D, Dikic I (2014). Ubiquitination in disease pathogenesis and treatment. Nat Med.

[52] Ksa B, Aw B, Mu B, Tima C, D P, Pdj A (2022). Development of a bi-layered cryogenic electrospun polylactic acid scaffold to study calcific aortic valve disease in a 3D co-culture model. Acta Biomater.

[53] Chen W, Zhou Y, Yang J, Liu F, Wu X, Sha Y (2020). Melatonin attenuates calcium deposition from vascular smooth muscle cells by activating mitochondrial fusion and mitophagy via an AMPK/OPA1 signaling pathway. Oxid Med Cell Longev.

[54] Carling D (2017). AMPK signalling in health and disease. Curr Opin Cell Biol.

[55] Garcia D, Shaw RJ (2017). AMPK: mechanisms of cellular energy sensing and restoration of metabolic balance. Mol Cell.

[56] Faubert B, Boily G, Izreig S, Griss T, Samborska B, Dong Z (2013). AMPK is a negative regulator of the Warburg effect and suppresses tumor growth in vivo. Cell Metab.

[57] Rodda SJ, McMahon AP (2006). Distinct roles for hedgehog and canonical Wnt signaling in specification,differentiation and maintenance of osteoblast progenitors. Development.

[58] Deberardinis RJ, Lum JJ, Hatzivassiliou G, Thompson CB (2008). The biology of cancer: metabolic reprogramming fuels cell growth and proliferation. Cell Metab.

[59] Gomel M, Lee R, Grande-Allen K (2018). Comparing the role of mechanical forces in vascular and valvular calcification progression. Front Cardiovasc Med.

[60] Shi J, Yang Y, Cheng A, Xu G, He F (2020). Metabolism of vascular smooth muscle cells in vascular diseases. Am J Physiol Heart Circ Physiol.

[61] Heinle H, Stowasser F, Betz E (1982). Metabolic changes in modified smooth muscle cells of rabbit carotid arteries. Basic Res Cardiol.

[62] Ma W, Sun X, Zhu Y, Liu N (2020). PDK4 promotes vascular calcification by interfering with autophagic activity and metabolic reprogramming. Cell death Dis.

[63] Phadwal K, Vrahnas C, Ganley I, MacRae V (2021). Mitochondrial dysfunction: cause or consequence of vascular calcification?. Front cell Dev Biol.

[64] Alesutan I, Moritz F, Haider T, Shouxuan S, Gollmann-Tepeköylü C, Holfeld J (2020). Impact of β-glycerophosphate on the bioenergetic profile of vascular smooth muscle cells. J Mol Med (Berl, Ger).

[65] Reiter AK, Bolster DR, Crozier SJ, Kimball SR, Jefferson LS (2005). Repression of protein synthesis and mTOR signaling in rat liver mediated by the AMPK activator aminoimidazole carboxamide ribonucleoside. Am J Physiol-Endocrinol Metab.

[66] Pinner S, Sahai E (2008). PDK1 regulates cancer cell motility by antagonising inhibition of ROCK1 by RhoE. Nat Cell Biol.

[67] Luo S, Wang H, Bai L, Chen Y, Chen S, Gao K (2021). Activation of TMEM16A Ca(2+)-activated Cl(-) channels by ROCK1/moesin promotes breast cancer metastasis. J Adv Res.

[68] Esposito D, Pant I, Shen Y, Qiao RF, Yang X, Bai Y (2022). ROCK1 mechano-signaling dependency of human malignancies driven by TEAD/YAP activation. Nat Commun.

[69] Shi D, Wu F, Mu S, Hu B, Zhong B, Gao F (2019). LncRNA AFAP1-AS1 promotes tumorigenesis and epithelial-mesenchymal transition of osteosarcoma through RhoC/ROCK1/p38MAPK/Twist1 signaling pathway. J Exp Clin Cancer Res.

[70] Wang Y, Wang H, Li C, Zhang J, Chu Z, Liu P (2022). CircTUBGCP3 contributes to the malignant progression of rectal cancer. Dig Dis Sci.

