# Supplementary figures and images for: Rho A/ROCK1 signaling-mediated metabolic reprogramming of valvular interstitial cells toward Warburg effect accelerates aortic valve calcification via AMPK/RUNX2 axis
Source: Cell Death Dis. 2023 Feb 11;14(2):108. doi: 10.1038/s41419-023-05642-1 (PMC9922265; doi:10.1038/s41419-023-05642-1)

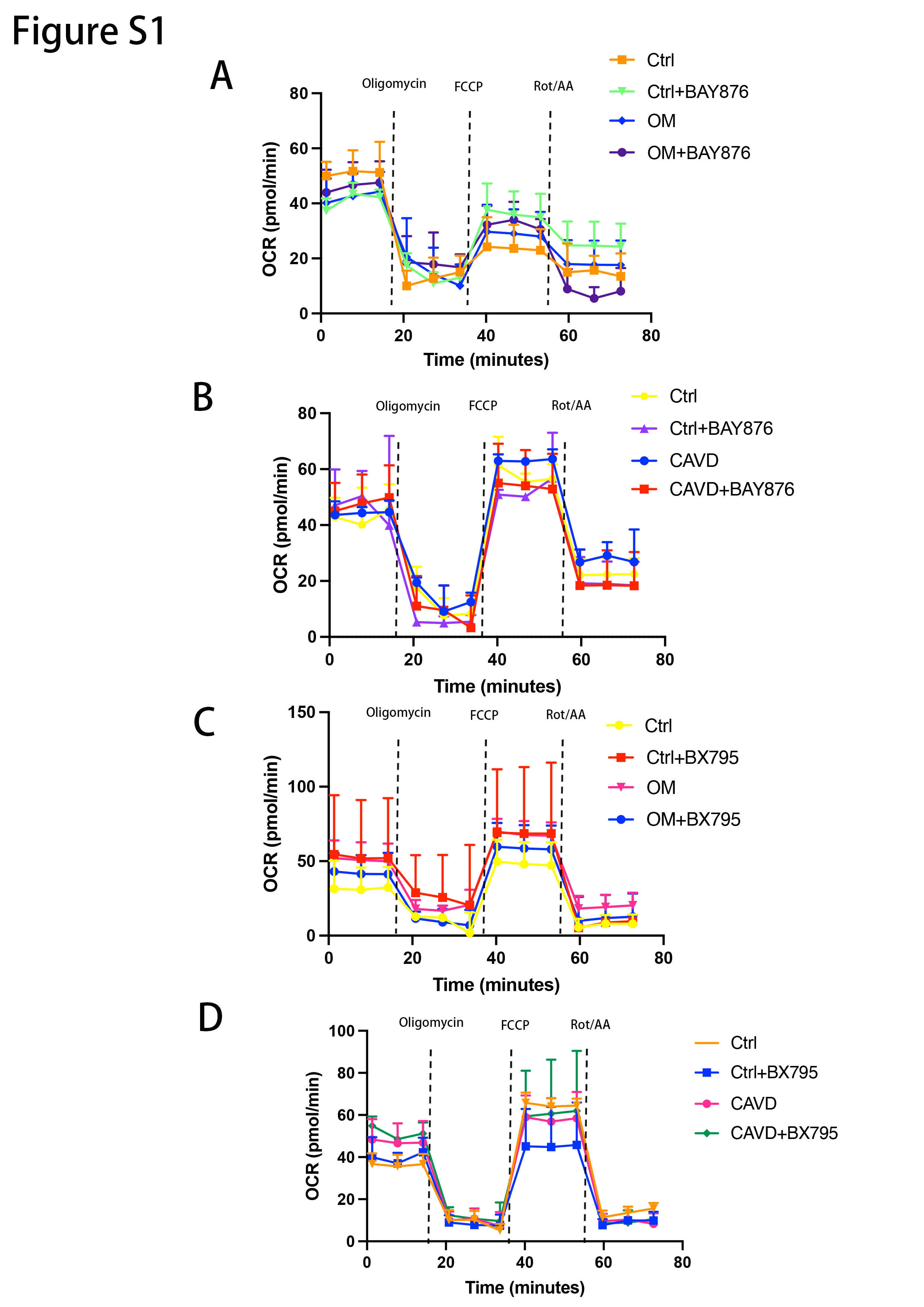

Supplement: Supplementary file 1 — Extended Figure S1 [file 41419_2023_5642_MOESM1_ESM.tif]
