# Supplementary material for: Rho A/ROCK1 signaling-mediated metabolic reprogramming of valvular interstitial cells toward Warburg effect accelerates aortic valve calcification via AMPK/RUNX2 axis
Source: Cell Death Dis. 2023 Feb 11;14(2):108. doi: 10.1038/s41419-023-05642-1 (PMC9922265; doi:10.1038/s41419-023-05642-1)
Supplement: Supplementary file 2 — Supplementary figure legends [file 41419_2023_5642_MOESM2_ESM.docx]

**Supplementary figure legends**

**Figure S1. A to D,** Seahorse profiles for oxygen consumption rate (OCR) for human VICs from CAVs, IP-OIM, or non-CAVs, cultured in the presence or absence of BAY876/BX795.
