# Supplementary material for: Rho A/ROCK1 signaling-mediated metabolic reprogramming of valvular interstitial cells toward Warburg effect accelerates aortic valve calcification via AMPK/RUNX2 axis
Source: Cell Death Dis. 2023 Feb 11;14(2):108. doi: 10.1038/s41419-023-05642-1 (PMC9922265; doi:10.1038/s41419-023-05642-1)
Supplement: Supplementary file 3 — Supplemental Table S2 [file 41419_2023_5642_MOESM3_ESM.docx]

| **Names** | **Sequences (5’-3’)** |
| --- | --- |
| *β-actin-F191* | GAAGAGCTACGAGCTGCCTGA |
| *β-actin-R191* | CAGACAGCACTGTGTTGGCG |
| *Homo-CBFA1-169F* | GGAGTGGACGAGGCAAGAGT |
| *Homo-CBFA1-169R* | AGGCGGTCAGAGAACAAACT |

**Supplementary Table S2. Sequences of primers used in RT-qPCR assay.**
