# Supplementary material for: Rho A/ROCK1 signaling-mediated metabolic reprogramming of valvular interstitial cells toward Warburg effect accelerates aortic valve calcification via AMPK/RUNX2 axis
Source: Cell Death Dis. 2023 Feb 11;14(2):108. doi: 10.1038/s41419-023-05642-1 (PMC9922265; doi:10.1038/s41419-023-05642-1)
Supplement: Supplementary file 4 — original data files [file 41419_2023_5642_MOESM4_ESM.pdf]

Figure 1

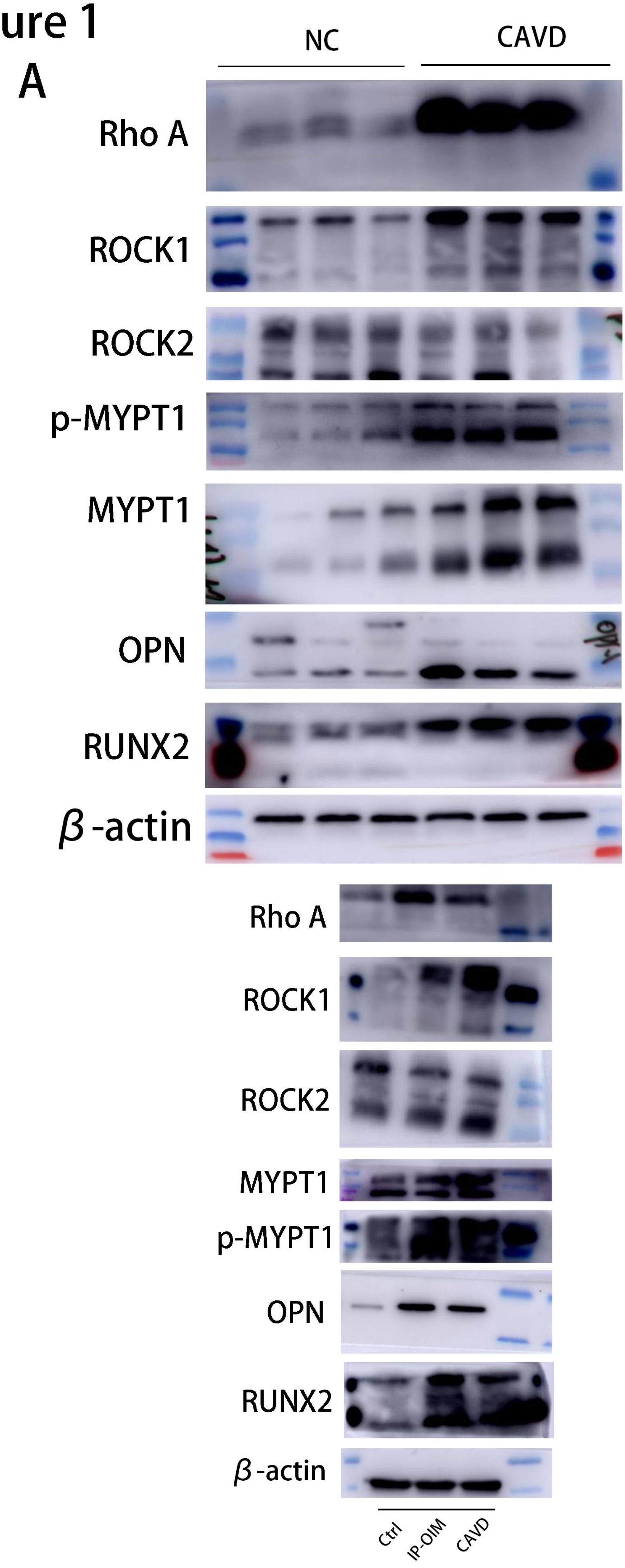
A
NC
CAVD
Rho A
ROCK1
ROCK2
p-MYPT1
MYPT1
OPN
RUNX2
β-actin
B
Rho A
ROCK1
ROCK2
MYPT1
p-MYPT1
OPN
RUNX2
β-actin
Ctrl
IP-OIM
CAVD

Figure2

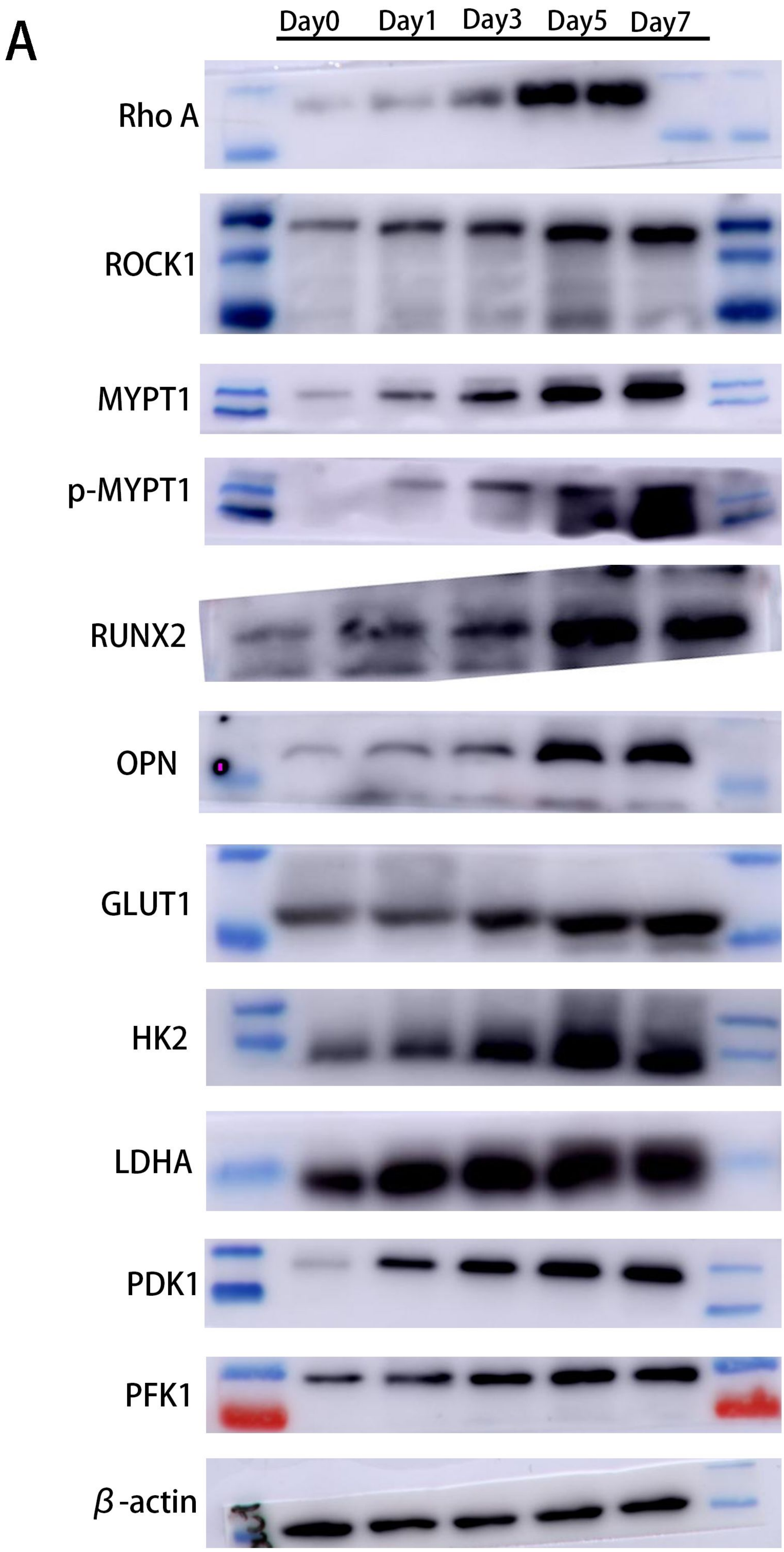
A
Day0
Day1
Day3
Day5
Day7
Rho A
ROCK1
MYPT1
p-MYPT1
RUNX2
OPN
GLUT1
HK2
LDHA
PDK1
PFK1
β-actin

Figure 3

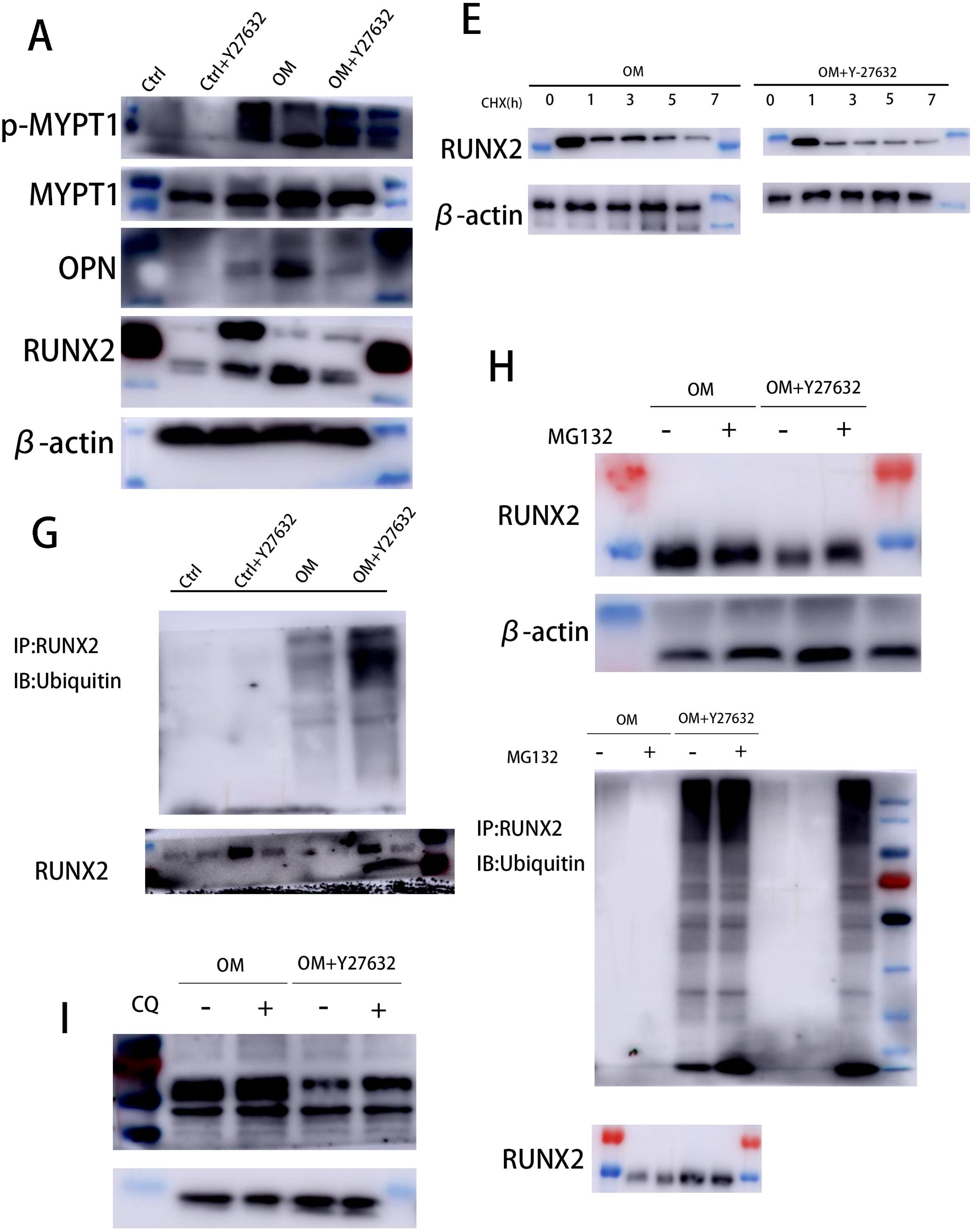
A
Ctrl
Ctrl+Y27632
OM
OM+Y27632
p-MYPT1
MYPT1
OPN
RUNX2
β-actin
E
OM
OM+Y-27632
CHX(h)
0 1 3 5 7
0 1 3 5 7
RUNX2
β-actin
G
Ctrl
Ctrl+Y27632
OM
OM+Y27632
IP:RUNX2
IB:Ubiquitin
RUNX2
H
OM
OM+Y27632
MG132
- + - +
RUNX2
β-actin
OM
OM+Y27632
MG132
- + - +
IP:RUNX2
IB:Ubiquitin
RUNX2
I
OM
OM+Y27632
CQ
- + - +

Figure 4

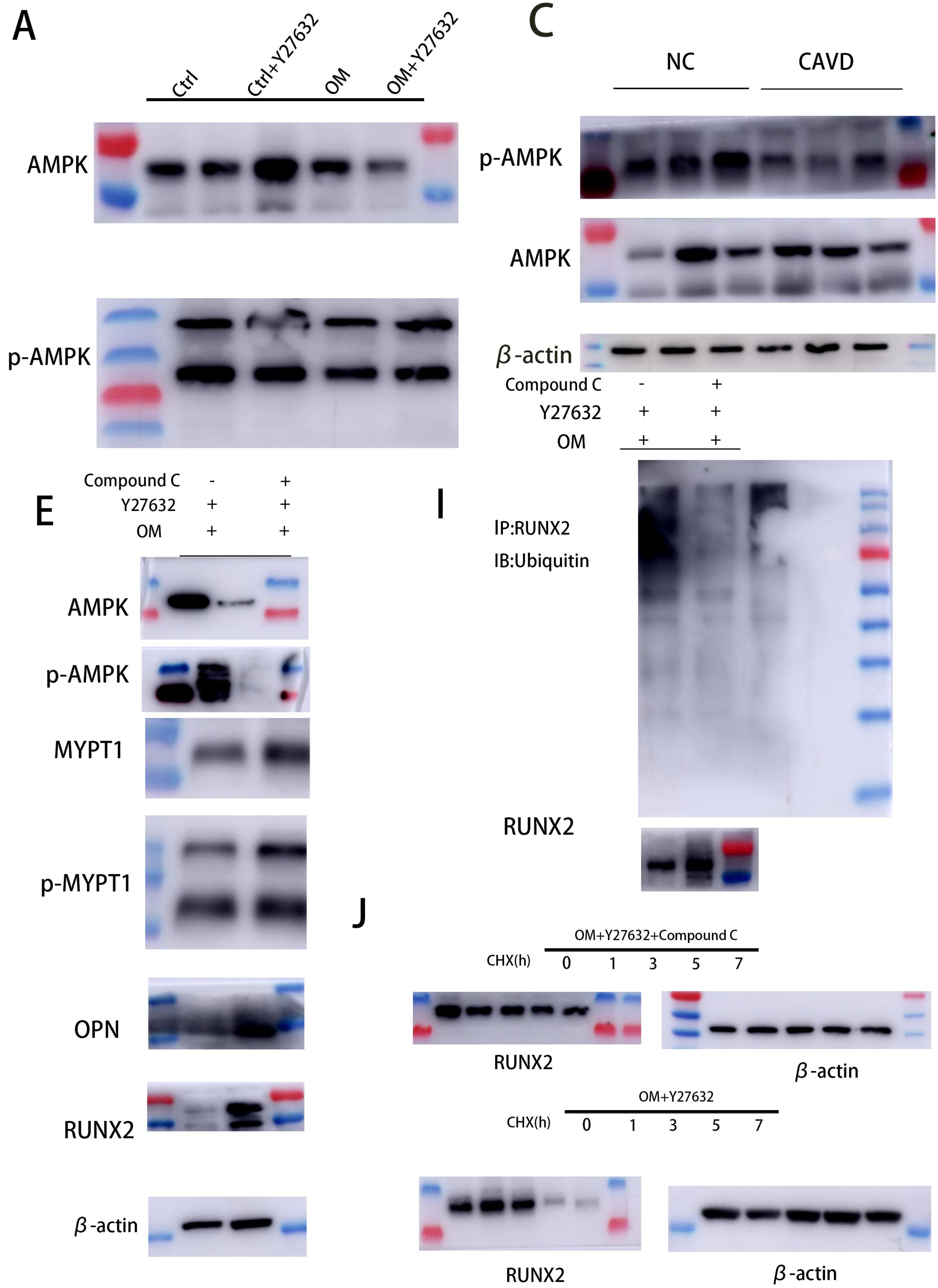
A
Ctrl
Ctrl+Y27632
OM
OM+Y27632
AMPK
p-AMPK
C
NC
CAVD
p-AMPK
AMPK
β-actin
Compound C - +
Y27632 + +
OM + +
E
Compound C - +
Y27632 + +
OM + +
AMPK
p-AMPK
MYPT1
p-MYPT1
OPN
RUNX2
β-actin
I
IP:RUNX2
IB:Ubiquitin
RUNX2
J
OM+Y27632+Compound C
CHX(h) 0 1 3 5 7
RUNX2
β-actin
OM+Y27632
CHX(h) 0 1 3 5 7
RUNX2
β-actin

Figure 5

A

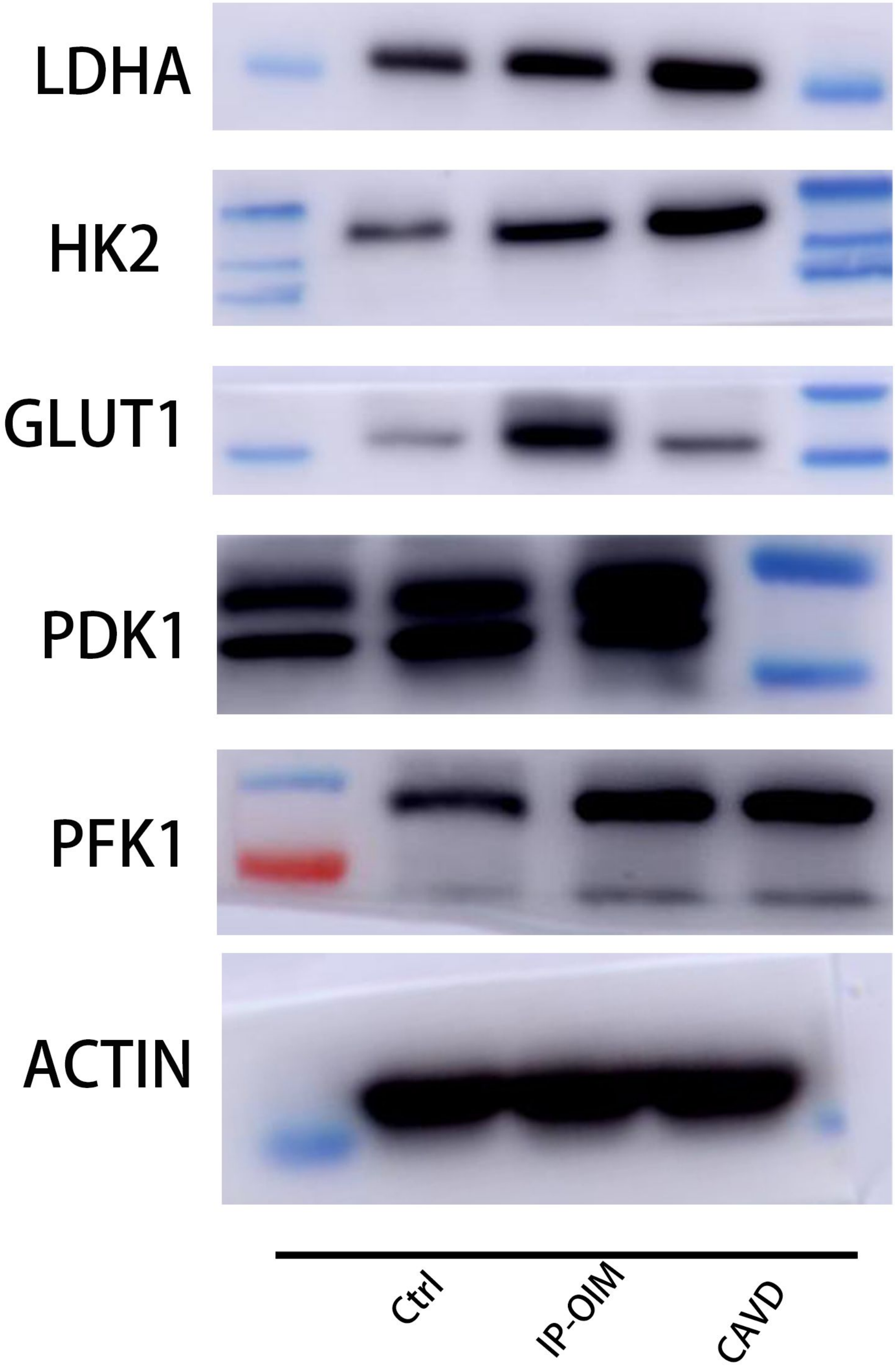

Figure 6

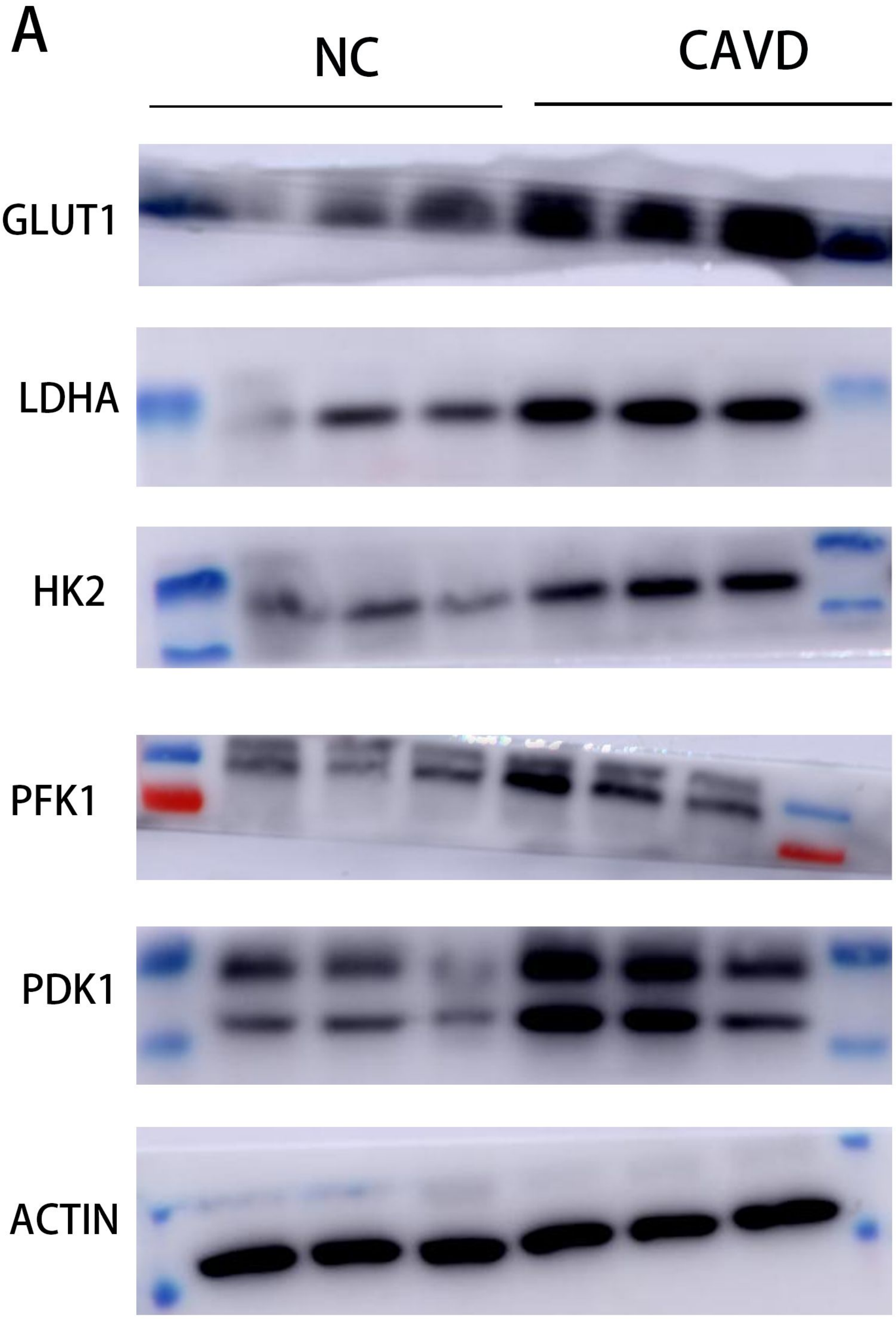

Figure 7

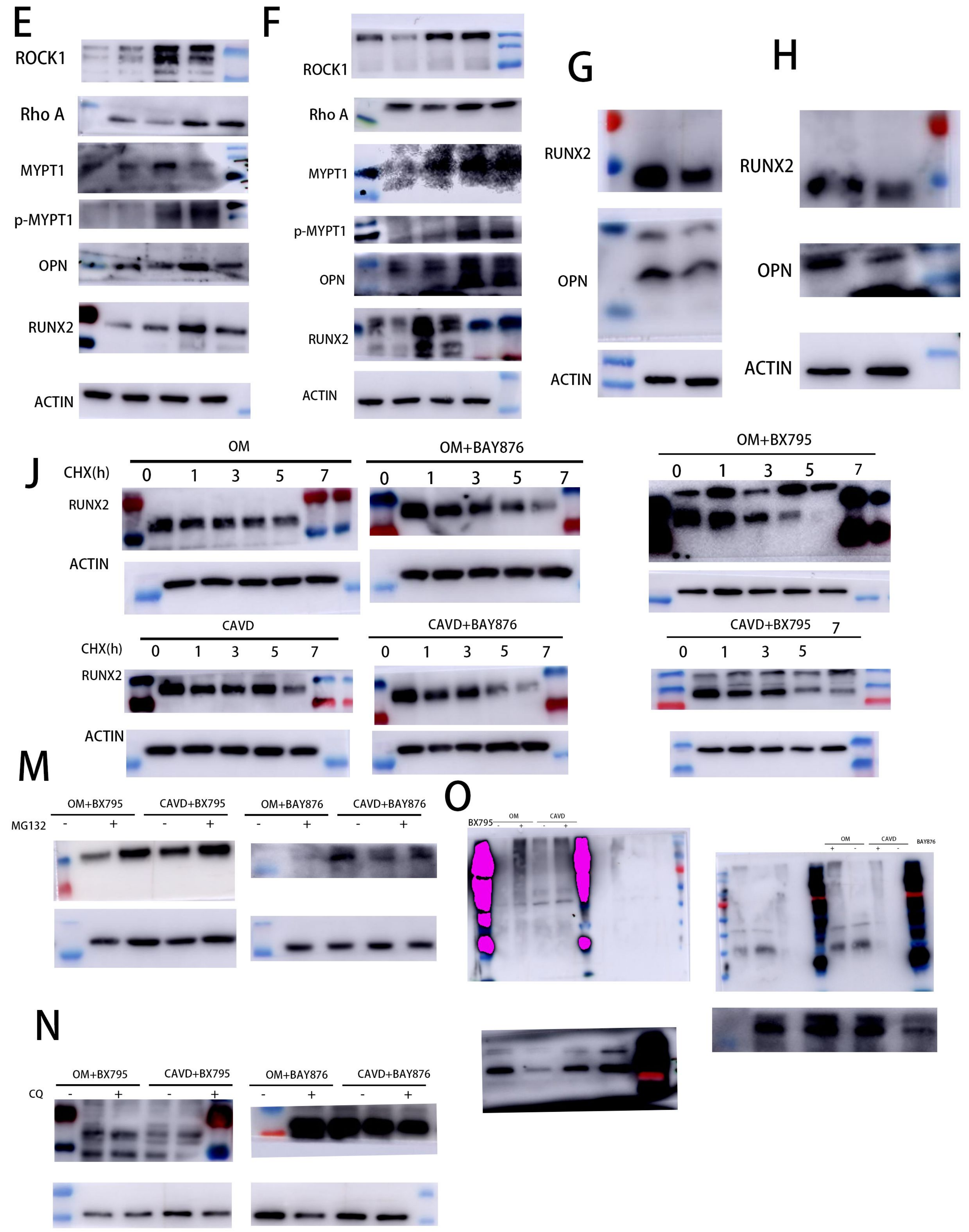
E
ROCK1
Rho A
MYPT1
p-MYPT1
OPN
RUNX2
ACTIN
F
ROCK1
Rho A
MYPT1
p-MYPT1
OPN
RUNX2
ACTIN
G
RUNX2
OPN
ACTIN
H
RUNX2
OPN
ACTIN
J
OM
OM+BAY876
OM+BX795
CHX(h)
0 1 3 5 7
RUNX2
ACTIN
CAVD
CAVD+BAY876
CAVD+BX795
CHX(h)
0 1 3 5 7
RUNX2
ACTIN
M
OM+BX795
CAVD+BX795
OM+BAY876
CAVD+BAY876
MG132 - + - + - + - +
O
BX795
OM
CAVD
BAY876
N
OM+BX795
CAVD+BX795
OM+BAY876
CAVD+BAY876
CQ - + - + - + - +

Figure 8

G

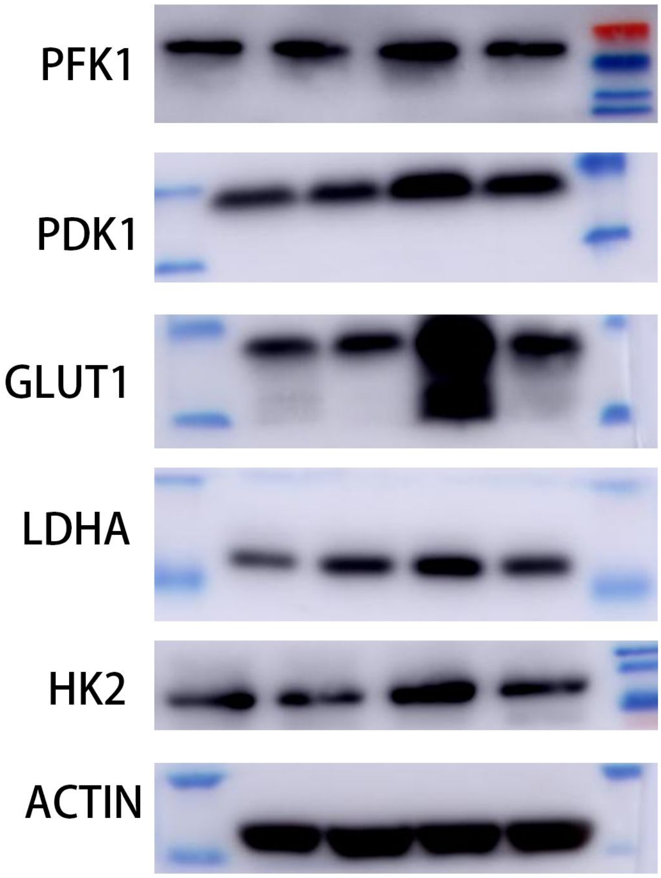
PFK1
PDK1
GLUT1
LDHA
HK2
ACTIN

H

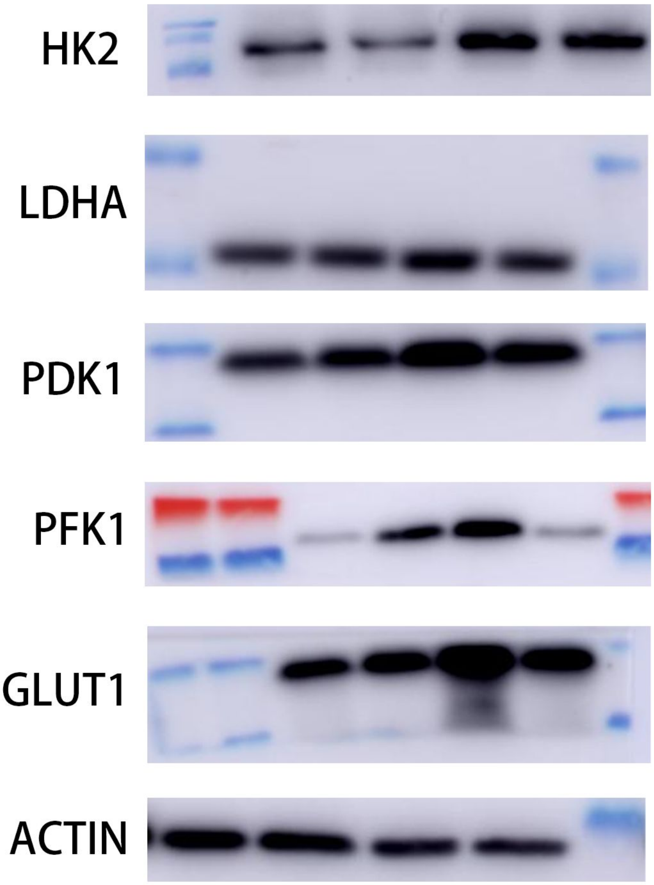
HK2
LDHA
PDK1
PFK1
GLUT1
ACTIN
